# Sex-biased transcriptomic landscapes in bipolar disorder: integrating neurobiology and clinical heterogeneity through cross-study meta-analysis

**DOI:** 10.1186/s13293-026-00870-4

**Published:** 2026-05-08

**Authors:** Omran Davarinejad, Mohammad-Taher Moradi, Arash Safarzadeh, Masumeh Jalalvand, Fatemeh Kazemisafa

**Affiliations:** 1 Clinical Research Development Center, Imam Khomeini and Mohammad Kermanshahi and Farabi Hospitals, Kermanshah University of Medical Sciences, Kermanshah, Iran; 2 Sleep Disorders Research Center, Health Policy and Promotion Institute, Kermanshah University of Medical Sciences, Kermanshah, Iran; 3 Department of Medical Genetics, Shahid Beheshti University of Medical Sciences, Tehran, Iran; 4 Medical Biotechnology, School of Medicine, Lorestan University of Medical Sciences, Khorramabad, Iran

**Keywords:** Bipolar disorder, Transcriptomic, Sex differences, Meta-analysis, Post-mortem brain, RNAseq

## Abstract

**Background:**

Bipolar disorder (BD) exhibits significant sex differences in its frequency, symptom presentation, and treatment response, suggesting distinct underlying neurobiological mechanisms. However, transcriptomic studies investigating these sex-specific pathways have been fragmented and underpowered.

**Method:**

We conducted the first meta-analysis of post-mortem brain RNA-seq data to delineate sex-related transcriptomic landscapes in BD. We integrated data from four public datasets (GSE80336, GSE80655, GSE202537, GSE42546) from GEO and Array Express, comprising an aggregate of 173 individuals (66 BD cases and 117 controls). After preprocessing and correcting for batch effects, sex-stratified expression analysis was performed using DESeq2. A meta-analysis was conducted with the metafor package to identify differentially expressed genes (DEGs) at an FDR < 0.05. We also performed functional enrichment, protein-protein interaction (PPI) network analysis, hub gene identification, regulatory network reconstruction, and supplementary quantitative analyses of sex-specific interaction effects.

**Results:**

Our results reveal striking differences in transcriptomic signatures between men and women with bipolar disorder, with the most pronounced changes occurring in the brain. A meta-analysis across brain regions identified 34 significantly dysregulated genes. In females, upregulated genes were enriched for hormonal signaling (FSHR pathway, G-protein signaling) and transcriptional/epigenetic regulation (GLIS1, neural plasticity). In males, upregulated genes were involved in synaptic calcium signaling (PDLIM5, dendritic spine regulation) and DNA mismatch repair pathways (PMS1). Analysis of the striatum identified 289 differentially expressed genes. The most significantly upregulated genes in females were implicated in immunity and synaptic plasticity, while the male-specific pattern pointed to alterations in basic cellular functions like structure, internal communication, and genetic regulation. A quantitative interaction analysis revealed a negligible correlation (*r* = -0.122) between disease effect sizes in females and males and identified one gene with opposing, sex-dependent dysregulation (MEF2C).

**Conclusion:**

This study provides robust evidence that bipolar disorder engages fundamentally distinct molecular pathways in males and females, underscoring the necessity of integrating sex as a biological variable in psychiatric research and advancing toward personalized therapeutic strategies.

**Supplementary Information:**

The online version contains supplementary material available at 10.1186/s13293-026-00870-4.

## Abstract (plain language summary)

Bipolar disorder is a mental health condition that affects mood, energy, and activity levels. It is well known that the disorder affects men and women differently. However, the biological reasons for these differences are not well understood.

In this study, we investigated these differences by analyzing gene activity in brain tissue from people with and without bipolar disorder. By combining data from several existing studies, we created a large dataset to see how the disorder’s biology differs between sexes.

We found that the biological basis of bipolar disorder is fundamentally different in men and women. The most striking differences were in a brain region called the striatum, which is involved in reward and motivation. In men, gene changes were related to basic cell functions like energy production. In women, the changes involved genes for brain cell communication and immune response.

This means the illness process differs between males and females. These findings help explain why symptoms vary by sex. Ultimately, this research suggests that doctors should consider sex as a crucial factor. Understanding these separate biological pathways could lead to better, more personalized treatments for everyone living with bipolar disorder.

**Supplementary Information:**

The online version contains supplementary material available at 10.1186/s13293-026-00870-4.

## Highlights

- We combined data from multiple brain studies to compare gene activity between men and women with bipolar disorder (BD).
- Men and women with BD show completely different patterns of gene activity in the brain. The most significant differences were found in the striatum, a brain region linked to reward and motivation.
- In men with BD, genes involved calcium homeostasis, synaptic vesicle release, and mitochondrial metabolism were more active.
- In women with BD, genes related to hormonal signaling, synaptic plasticity, and immune regulation were more active.
- Our quantitative interaction analysis revealed that disease effect sizes are largely independent between sexes (*r*=-0.122), and we identified genes with opposing patterns of dysregulation.
- Our findings strongly suggest that bipolar disorder develops and presents differently in men and women at a biological level, highlighting the critical need for sex-specific approaches in research and treatment.

**Supplementary Information:**

The online version contains supplementary material available at 10.1186/s13293-026-00870-4.

## Background

Bipolar disorder is a chronic, recurrent condition characterized by marked fluctuations in mood, which include episodes of depression and mania, and have a substantial impact on the individual’s functioning and quality of life [1, 2]. Epidemiologically, the lifetime prevalence of the disorder is 2%–4% with an associated heavy burden on affected individuals, families, and societies [3]. Mortality related to bipolar disorder is high, with the mean age of premature death among individuals with bipolar disorder and approximately 10–15 years and 9–14 years for women and men, respectively [4].

Neurobiological impairments in bipolar disorder include amygdala and anterior paralimbic cortex (including ventral prefrontal cortex, insula, and temporal poles) dysfunction; these regions are important in the regulation of emotion [5]. Structural and functional alterations of the brain could be observed mainly in the prefrontal cortex, temporal cortex, cingulate gyrus, amygdala, hippocampus, and thalamus [6, 7]. Namely, diminished prefrontal gray matter volume and activity compromise executive function and mood regulation [8], and elevated amygdala activity throughout mood episodes, alongside volume loss, impairs emotional processing [9]. These disturbances of the principal networks of emotion and cognition in the brain are the basis for BD, with all its mood instability and dysregulation.

Bipolar disorder results from a combination of inherited risks, life experiences, and changes in brain chemistry and structure (8). Increased pro-inflammatory cytokine levels (e.g., IL-6, TNF-alpha) in mood states have been shown to reflect an ongoing inflammation, potentially influencing brain functioning and neuro-progression [9–11]. This pro-inflammatory condition is also linked with oxidative stress and mitochondrial dysfunction, leading to the compromised health of neurons of mood-regulating brain regions [9, 12], and altered monoamine and glutamatergic signaling that couples these processes with disturbances in neural circuits [11, 12].

Epidemiological studies consistently report sex differences in bipolar disorder presentation and course. Women demonstrate higher prevalence of bipolar II disorder, more depressive episodes, and rapid cycling, with a later age of onset compared to men. Conversely, men exhibit more manic episodes with mixed features [13–15] and have higher rates of substance use disorders [16].

Transcriptomic studies now present sex-specific divergence at a molecular level, with male patients having up-regulated glial/glutamate gene expression (e.g., *MBP*,* PLP1*) in the prefrontal cortex during psychosis [17], whereas females show reduced synaptic gene expression (e.g. *SCN2A*) and activated immune pathways (e.g. *C3*) [18]. Yet despite this accumulation of sex-specific biology emerging from the literature, molecular studies to date are fragmented and significantly underpowered. Single transcriptomic surveys do not tend to be sufficiently targeted or powered for detecting sex interaction effects.

Beyond the identification of differentially expressed genes, understanding the regulatory mechanisms that drive these sex-specific transcriptional changes is crucial. MicroRNAs (miRNAs) [19] and transcription factors (TFs) [20] represent two major layers of gene regulation that have been implicated in BD pathophysiology and sexual dimorphism in the brain [20]. miRNAs can fine-tune neuronal plasticity, stress responses, and immune function—all processes implicated in BD—[21] while TFs orchestrate broad transcriptional programs that may differentially impact male and female brains [20]. Therefore, mapping these regulatory networks provides mechanistic insights into how sex-specific molecular signatures in BD emerge and may reveal novel upstream controllers and therapeutic targets.

With this clinical-molecular disparity in mind, we undertook the first cross-study meta-analysis of RNA-seq data stratified by sex to bridge the gap between clear clinical heterogeneity and poorly understood molecular mechanisms. We predicted the greatest sex-differentiated signal in striatal and limbic regions, which are nodes of reward and emotional processing. We had four specific objectives: (1) to describe sex-specific transcriptional profiles across brain regions; (2) to identify sex-common profiles disrupted in BD; (3) to identify emerging sex-distributed biomarker candidates; and (4) to discover sex-divergent molecular pathways for targeting with new therapeutics.

## Methods

### Workflow of the analytical approach

To investigate sex-specific molecular mechanisms in bipolar disorder (BD), we employed a comprehensive bioinformatics pipeline identifying differentially expressed genes (DEGs) from RNA-seq data, followed by PPI network construction, pathway enrichment, regulatory network analysis, and protein-chemical exploration. Most analyses were performed in R version 4.4.1 [22], with packages and versions detailed in Supplementary Table 1 (S1).

### Data retrieval and preprocessing

Our study adhered to Preferred Reporting Items for Systematic reviews and Meta-Analyses (PRISMA guidelines) [23] We systematically retrieved RNA-seq datasets from Gene Expression Omnibus (GEO) [24] and Array Express [25] up to 25 April 2025 using the search terms detailed below.

- GEO query: bipolar [All Fields] AND “bipolar disorder“[All Fields] AND “Expression profiling by high throughput sequencing“[Filter] (66 results).
- Array Express query: “bipolar disorder” (48 results).

**Fig. 1:**
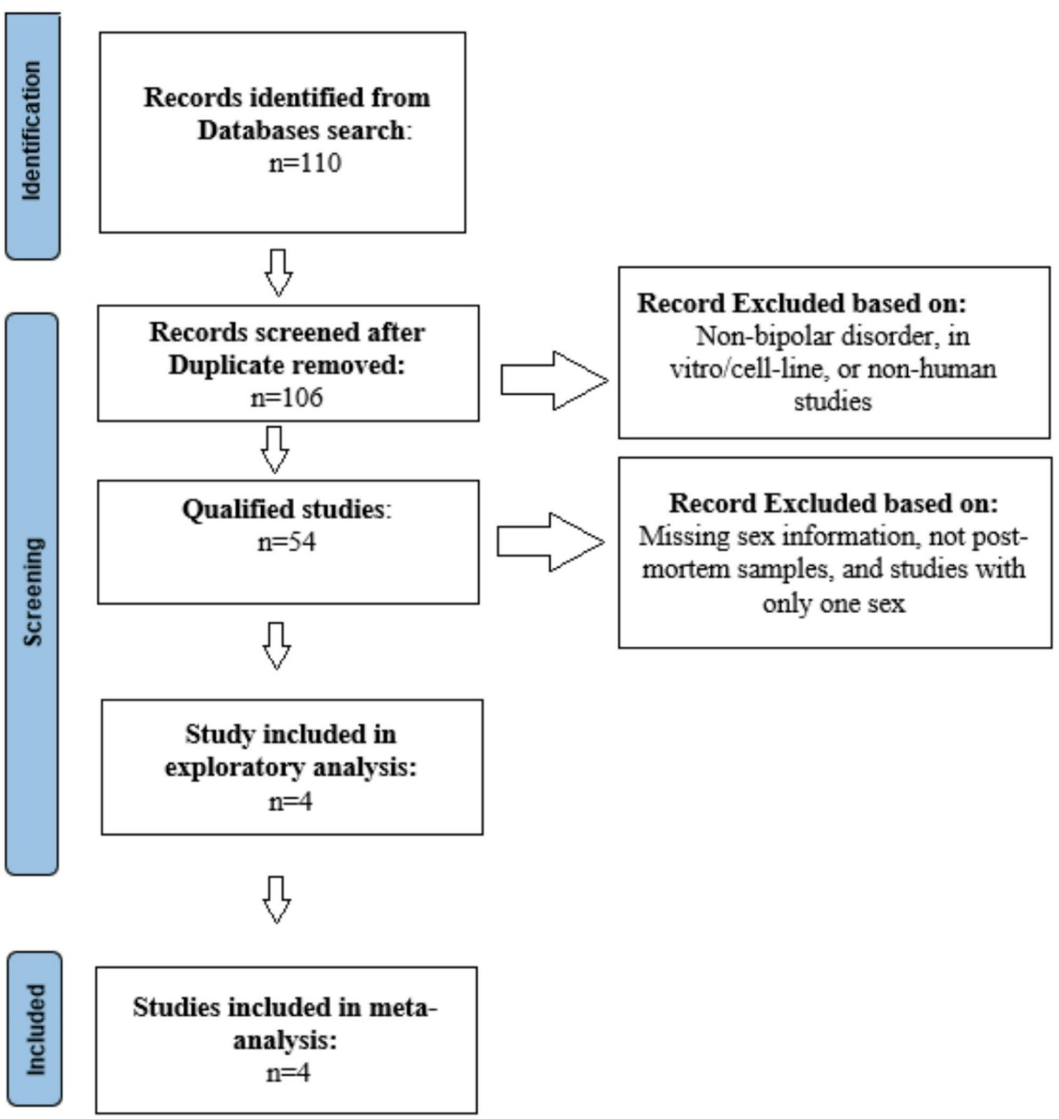
PRISMA Flow Diagram of Study Selection for the Cross-Study Meta-Analysis

Study screening and selection were performed independently by two reviewers; conflicts were resolved through consensus or by a third senior researcher. This initial search yielded 110 unique records. After a multi-stage screening process against our predefined inclusion and exclusion criteria (detailed in the PRISMA flow diagram, Fig. 1), 4 RNA-seq datasets (GSE80336, GSE80655, GSE202537, GSE42546) were included in the final meta-analysis. The primary reasons for exclusion of the 106 records were: non-human studies, use of cell lines, absence of RNA-seq data, lack of sex-stratified metadata, or insufficient sample size (minimum sample size > 3 per group for both sexes).

### Differential expression analysis

Data preprocessing.

Before differential expression analysis, we carried out extensive preprocessing of the RNA-seq data to yield robust and interpretable results:

#### Gene annotation

Raw gene identifiers were converted to official HUGO Gene Nomenclature Committee (HGNC) symbols [25] by using the biomaRt package (version 2.62.1) [26], which soft-enforces consistency across datasets.

#### Normalization

Raw gene-expression data were preprocessed to remove entries with missing/null (gene symbols), and mean gene expression values of the duplicate genes were calculated. Library size normalization and variance stabilization were done with DESeq2’s variance-stabilized transformation (VST). Post-normalization boxplots between healthy and patient groups revealed that effective technical processing was achieved, demonstrating homogeneous variance across the expression range, and the data distribution for parametric tests was suitable.

#### Sex-stratified DEG Identification

We utilized a stringent two-stage analysis with DESeq2 (version 1.46.0) [26]. For the pan-brain analysis, which included datasets with samples from multiple brain regions per subject (GSE202537 and GSE80655), the differential expression analysis was performed on all samples combined, not separately by region. To account for the non-independence of multiple samples originating from the same individual and avoid pseudoreplication, Specifically, for each subject with multiple brain region samples, we aggregated the gene expression counts across all available brain regions by summing the raw counts for each gene. This aggregation approach generated a single, representative expression profile per individual, ensuring that each subject contributed only one data point to the differential expression analysis. This method preserves the overall transcriptional signal while eliminating the artificial inflation of sample size that would occur if multiple region samples from the same individual were treated as independent replicates.

Then we generated primary contrasts of interest (separately for each sex): [1] BD. female vs. Control. female and [2] BD.male vs. Control. male with DESeq2’s negative binomial model. While DESeq2 can directly estimate interaction effects in a single-stage generalized linear model, we employed this two-stage approach for specific reasons pertinent to our meta-analytic design. The integration of four heterogeneous datasets from different brain regions necessitates a robust framework for handling between-study variance. Analyzing each dataset separately and then meta-analyzing the effect sizes is a standard and validated method for such designs. This approach also provides clear, interpretable lists of sex-specific differentially expressed genes, which are essential for our downstream functional analyses (e.g., PPI, pathway enrichment).

To formally identify sex-by-diagnosis interaction effects, we calculated an interaction log2 fold change (LFC) for each gene as: LFC_interaction = LFC(BD vs. Ctrl in Females) - LFC(BD vs. Ctrl in Males). This difference-in-differences approach is mathematically equivalent to estimating the β₃ coefficient in a linear model of the form: Expression ~ β₀ + β₁×Sex + β₂×Diagnosis + β₃×(Sex×Diagnosis) + ε, where a significant β₃ indicates a differential disease effect between sexes. A formal interaction analysis was used to identify sex-biased specific effects of disease: (BD. female - Control. female) - (BD. male - Control. male). Genes were defined as female-increased DEGs (LFC > 0, FDR < 0.05) and male-increased genes (LFC < 0, FDR < 0.05). This classification captures all relevant scenarios: upregulation in one sex only, downregulation in one sex only, or opposite-direction effects.This approach is particularly effective at capturing genes with sexually divergent responses, where the disease effect operates in opposite directions. Such opposite-direction effects generate the strongest interaction signals (largest absolute LFC_interaction values). For example, in our data, the gene PBRM1 exhibited a strong male-biased interaction in one cohort (Study 3: LFC_interaction = -0.916) but female-biased interactions in others, with the meta-analysis yielding a significant overall female-biased effect (LFC_interaction = + 0.368, FDR = 0.048).

Limbic system (emotion/memory), striatal system (motor/reward), and combined pan-brain analyses were carried out separately. The results of this interaction analysis are reported in detail in Sect.  3.7 of our Results.

#### Cross-dataset DEG consensus

Based on separate differential expression analyses on each dataset using DESeq2 [26], we implemented a consensus strategy across datasets to identify overlapping differentially expressed genes (DEGs). We identified DEGs shared across all four datasets (across all brain regions, the striatal system, and the limbic system) using the VennDiagram package (version 1.7.3) [27] and the intersect() function. The common set of genes shared across studies was extracted from the normalized counts, aligned based on Ensembl gene identifiers, and subsequently used as input for a two-level meta-analysis. This workflow ensured that our downstream analyses prioritized stable and reproducible expression changes associated with bipolar disorder.

### Meta-analysis

We employed a two-tiered meta-analytic strategy to balance biological interpretability with statistical power. First, to identify a high-confidence core of genes consistently altered across heterogeneous brain regions, we focused the pan-brain (combined) meta-analysis on the 5,130 genes present in all four datasets (see Sect.  2.3.5). Second, for regionally focused analyses (striatal and limbic systems), where tissue heterogeneity was reduced, we performed meta-analyses on all genes common to the studies within each specific system (e.g., 17,155 genes for the striatal system) to maximize sensitivity for detecting region-specific effects.

All meta-analyses were performed using the metafor package (version 4.8-0) [27], employing Restricted Maximum Likelihood (REML) [28]. A REML random-effects model was chosen a priori due to the anticipated clinical and technical heterogeneity across independent studies, as it provides superior performance in handling between-study variance, particularly for smaller sample sizes. Heterogeneity was quantified using I² statistics and Cochran’s Q-test. Meta-analyses were conducted separately for: Limbic system, Striatal system, and Combined analysis (all brain regions) to identify overarching sex-biased signatures. Funnel and forest plots were generated for each analysis, using log2 fold changes (LFC) as effect sizes and their standard errors (SE) as precision metrics, to evaluate potential bias and heterogeneity [29]. To assess the robustness of the meta-analysis results, we performed leave-one-out sensitivity analyses for each significant gene. Gene-disease associations with BD were assessed through the Open Targets Platform (release 24.06) [30], leveraging its curated knowledge base of disease-gene relationships.

### Functional enrichment and PPI networks

We performed Over-Representation Analysis (ORA) [31] with the clusterProfiler package (v4.14.6) [32], and to find enriched biological functions in three gene sets (a) female-upregulated (LFC > 0), (b) male-upregulated (LFC < 0), (c) all DEGs. In the case of the enrichment significances of the biological process Gene Ontology terms (BP) all nominal and adjusted p-values have been used. The significance threshold was set at an FDR-adjusted p-value < 0.05.

### Construction of PPI network and identification of Hub genes

The DEGs screened out of the male and female groups were used to build sex-specific protein-protein interaction (PPI) networks by Cytoscape (version 3.10.3). Known and predicted interactions were obtained from the STRING database.

For network construction, we applied a region-specific approach to threshold selection:

For the striatal system analysis, which yielded a substantial number of significant genes (127 female-upregulated, 162 male-upregulated), we used the default STRING confidence threshold (minimum required interaction score = 0.4) to ensure high-confidence interactions.For the pan-brain analysis, where the number of significant genes was substantially reduced following aggregation (15 female-upregulated, 19 male-upregulated), the default threshold yielded no consensus hub genes. Therefore, for exploratory purposes only, we lowered the minimum required interaction score to 0.15 for this specific analysis to identify potential key nodes despite the smaller gene sets. This adjustment is explicitly noted in the corresponding results section, and these findings should be interpreted with caution.

Densely connected regions in the network (clusters) were detected using the MCODE plugin [33] for each of the networks at the following parameters: degree cutoff = 2, node score cutoff = 0.2, k-core = 2, and max depth = 100. We found biologically relevant clusters by using MCODE analysis, with selection of high-scoring clusters according to three main topological parameters: [1] node color intensity (degree centrality) [2], node size (betweenness centrality), and [3] edge thickness (edge betweenness). This multi-parameter analysis ensured that the most functionally relevant network modules for each sex were reliably detected.

Then, the hub genes in each sex-specific network were screened by four centrality methods provided by the cytoHubba plugin [34] (MCC, Degree, Betweenness, and Closeness). For every such measure, the top 10-ranked genes were selected. The common genes in all four CENTs lists were determined as the consensus hub genes. (screened using a Venn diagram approach). This method identified sex-dependent hub genes with strong network significance.

### Gene regulatory networks (GRN) analysis

To reveal sex-specific regulatory motifs driving the observed transcriptomic differences, we constructed GRNs by integrating transcription factor (TF) binding profiles from JASPAR [35], and experimentally validated miRNA-target interactions from miRTarBase(version9.0) [36]. This approach allows us to identify key regulatory nodes that might orchestrate the sex-specific transcriptional programs in BD. We prioritized regulators based on their connectivity in the networks, as highly connected nodes (hubs) are more likely to exert significant influence on gene expression patterns. Regulatory relationships were combined and presented for the analysis by NetworkAnalyst [37], which permitted global analysis of TF-gene interactions in sex-specific DEGs and miRNA-target relationships in sex-specific DEGs.

### Protein-chemical prediction

We built Protein-chemical interaction networks using NetworkAnalyst [37] to find the possible therapeutic compounds acting on our significant sex-specific genes. Protein/chemical interaction data were obtained from Comparative Toxicogenomics Database (CTD) [38] and used to identify compounds interacting with sex-specific disease mechanisms.

### Pathway analysis

Functional annotation of differentially expressed sex-specific genes was conducted using Enrichr [39]. Pathway enrichment was evaluated against KEGG, WikiPathways, Reactome, BioPlanet, and BioCarta databases with an FDR < 0.05 value.

### Analysis of baseline sex differences

To determine if sex-specific disease effects were influenced by pre-existing baseline sex differences, we performed a complementary meta-analysis. For each of the four datasets, a differential expression analysis was conducted comparing healthy male controls vs. healthy female controls using DESeq2. The resulting log2 fold changes (LFC) and standard errors (SE) for each gene were then combined in a meta-analysis using the metafor package with a random-effects model (REML). Genes with an FDR-adjusted p-value < 0.05 were considered to have significant baseline sex differences. The final list of baseline sex-difference genes was then compared to our lists of sex-specific disease genes (Supplementary Table S29).

### Supplementary quantitative analysis of sex-specific interaction effects

To quantitatively assess the divergence of transcriptional responses between sexes using threshold-free methods, we performed additional analyses on the effect sizes from individual studies. First, we extracted log2 fold change (LFC) estimates and their standard errors for BD vs. control comparisons, separately for males and females, from all four datasets. For each gene present across all datasets, we calculated the average female-specific LFC and average male-specific LFC across studies. The Pearson correlation coefficient between these average effect sizes was computed to evaluate the overall concordance of disease-associated expression changes between sexes. Second, to formally test for sex-by-diagnosis interaction effects, we performed a gene-wise meta-analysis of the interaction term. For each gene, a random-effects model was fitted using the metafor package to estimate the difference between the female-specific effect size (β_female) and the male-specific effect size (β_male). The significance of this interaction term (β_female - β_male) was assessed, and p-values were adjusted for multiple testing using the Benjamini-Hochberg (FDR) procedure. Genes with an FDR-adjusted p-value < 0.05 for the interaction term were considered to have significant sex-dependent disease effects. Visualization included a scatter plot of average effect sizes and a histogram of the interaction term distribution.

## Result

To assess differences in BD according to sex, we conducted a systematic review and three meta-analyses of transcriptomic studies with data on patient sex from GEO [24] and Array Express databases [25]. The limbic system (2 studies), striatal system (3 studies), and combined pan-brain analysis (4 studies) were separated in the meta-analyses. Finally, we investigated the biological implications of the three meta-analysis results through using a set of different functional profiling techniques: over-representation analysis (ORA), construction of protein–protein interaction (PPI) networks, and analysis of regulators such as transcription factors (TFs) interactions, miRNA interactions, protein-chemical interactions, and Pathway Analysis.

### Study search and selection

We included 4 studies that were met our inclusion and exclusion criteria (Fig. 1), containing 173 subjects, and controls (107 control and 66 BD, respectively) that was isolated from the Dorsal striatum, anterior cingulate cortex, dorsolateral prefrontal cortex and nucleus accumbens, NAc, Caudate, Putamen and hippocampus dentate gyrus (DG) granule cells (Table 1). The final analytical cohort consisted of 66 BD patients (36 Male, 30 Female) and 107 healthy controls (70 Male, 37 Female).

**Table 1:** Characteristics of RNA-seq datasets included in the meta-analysis

Study accession	Platform	Brain region	BD patients (male)	BD patients (female)	Healthy control (male)	Healthy control (female)	Total samples	Publication
**GSE80336**	Illumina HiSeq 2000 (Homo sapiens)	DC	6	12	7	11	36	[75]
**GSE80655**	Illumina HiSeq 2000 (Homo sapiens)	ACC, DPC, and NAc	16	8	21	3	48	[76]
**GSE202537**	Illumina NextSeq 500 (Homo sapiens)	NAc, C, and P	5	3	25	11	44	[77]
**GSE42546**	AB SOLiD 4 System (Homo sapiens)	DG, GC, and HP	9	7	17	12	45	[78]
**Total**			**36**	**30**	**70**	**37**	**173**	

BD: Bipolar Disorder, DS: dorsal striatum, NAc: Nucleus Accumbens, DG: Dentate Gyrus, GC: granule cells, HP: hippocampus, ACC: anterior cingulate cortex, DPC: dorsolateral prefrontal cortex, C:Caudate, P:putamen

### Individual analysis

Principal Component Analysis (PCA) was performed on the four datasets after aggregating multiple brain region samples per subject in GSE202537 and GSE80655. The analysis revealed no significant batch effects, confirming that the aggregation approach successfully mitigated technical variability associated with multiple brain regions per individual. The data were therefore deemed suitable for downstream differential expression analysis without additional batch correction.

### Meta-analysis and functional enrichment

#### Meta-analysis in all studies

**Fig. 2:**
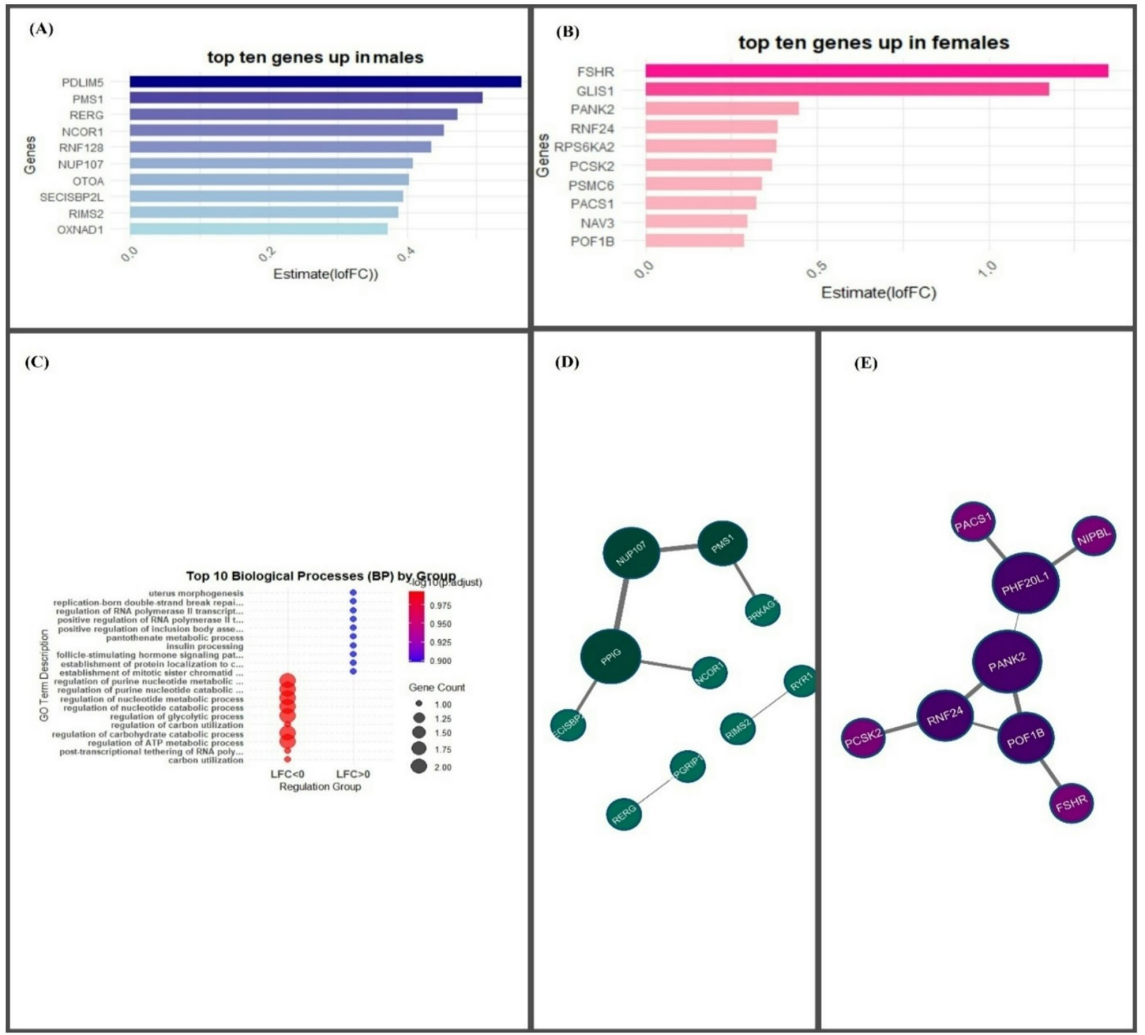
Sex-Biased Transcriptomic Signatures in the Pan-Brain Meta-Analysis. (**A**, **B**) The top ten significantly upregulated genes in (**A**) males and (**B**) females, ranked by absolute log2 fold change (LFC), identified from the meta-analysis of all brain regions. Error bars represent the 95% confidence interval for the LFC estimate. (**C**) Dot plot of significantly enriched Gene Ontology (GO) Biological Process terms for female-upregulated (LFC > 0) and male-upregulated (LFC < 0) gene sets. Dot size represents the gene ratio (number of significant genes in the term divided by the total genes in the term); color intensity represents the statistical significance (-log10(adjusted p-value)). (**D**, **E**) Protein-protein interaction (PPI) networks of differentially expressed genes for (**D**) males and (**E**) females. Networks were generated in Cytoscape; the top functional module from each, as identified by MCODE clustering, is shown. Node color intensity corresponds to degree centrality; node size corresponds to betweenness centrality

Next, we performed a meta-analysis across all of the studies with the 5130 common genes obtained from a Venn diagram that included all datasets. Our findings showed sex-specific gene dysregulation in multiple brain regions in BD. In the whole-brain meta-analysis, 34 genes were significantly dysregulated (FDR < 0.05), including 15 that were female-upregulated (LFC > 0) and 19 that were male-upregulated (LFC < 0). Importantly, this classification captures diverse biological scenarios beyond simple upregulation in one sex, including genes downregulated specifically in one sex or showing opposite-direction effects across sexes (Supplementary Table S29). Leave-one-out sensitivity analysis confirmed the robustness of these findings (Supplementary Table S3). The bar chart for the top ten genes in females and males by log fold change and adjusted p-value is shown in Fig. 2 (A, B). Of these 34 genes, we found 10 (29.4%) that have previously been associated with bipolar disorder, according to the Open Targets database (Table 2), providing genetic validity to our transcriptomic results.

**Table 2:** Significantly impacted genes detected in the meta‑analysis of expression data from all studies (Significantly impacted genes grouped by LFC value. Genes with a documented association with BD in the OpenTargets database are highlighted in bold)

Direction of increment	Significant genes
LFC > 0	*NIPBL*,* OR9Q1*,* PANK2*,* RPS6KA2*,* PCSK2*,* RNF24*, ***PSMC6***, ***NAV3***, *PHF20L1*,* MRRF*,* NLRC4*, ***POF1B***, *GLIS1*, ***PACS1***, *FSHR*
LFC < 0	*SECISBP2L*, ***RCHY1***, ***OTOA***, *NUP107*,* POLR3F*,* RERG*,* PMS1*,* OXNAD1*,* NCOR1*,* PXMP4*,* RIMS2*, ***RPGRIP1L***, ***PDLIM5***, *RNF128*, ***ROR1***, *RYR1*,* SCAMP1*, ***PPIG***, *PRKAG1*

Key genes, grouped by their log2 fold change (LFC), with established BD associations (from OpenTargets) highlighted in bold

In females, the most significantly upregulated genes were involved in key signaling pathways, including FSHR (follicle stimulating hormone receptor) and GLIS1(GLIS family zinc finger 1). In contrast, male-upregulated genes were PDLIM5(PDZ and LIM domain 5) and PMS1 (mismatch repair system component). The complete list of the top ten upregulated genes for each sex, along with their full annotations, is provided in Fig. 2(A, B). Also, full meta-analysis results for all 34 DEGs, including Log2FC, 95% confidence intervals, and heterogeneity statistics (I²), are provided in Supplementary Table S2.

#### Over-represented functions and signaling pathways in all studies

ORA (Cluster Profiler package) of the significant genes indicated that significant enrichment in several key biological pathways whose expression patterns are regulated in a sex-dependent manner. Specifically, genes with increased expression in female samples (LFC > 0) were mainly involved in processes related to uterine morphogenesis and the follicle-stimulating hormone (FSH) signaling pathway. (Fig. 2C).

Enrichment analysis comparing male and female up-regulated genes was performed using several databases (KEGG, WikiPathways, Reactome, BioPlanet, Elsevier, and BioCarta) to identify significant pathways with an adjusted p-value < 0.05. The most enriched pathways in males were Reversal of Insulin Resistance by Leptin Homo sapiens h leptin Pathway and Single-Strand Mismatch. For females, the most relevant pathways were Proteins Involved in Primary Ovarian Insufficiency and alpha-Cell to beta-Cell Interconversion (Hypothesis) Full results are shown in Supplementary Tables 4, 5(S4-S5).

#### Protein–protein interaction networks and hub genes identification in all studies

The top cluster identified from the PPI network of males and females in all studies is shown in Fig. 2 (D, E). The consensus hub gene analysis for the all regions of brain revealed distinct key regulators for each sex. In males, the hub gene was *PPIG*. In females, the consensus hub genes were *PHF20L1* and *PANK2*. It is important to note that these findings are exploratory and should be interpreted with caution due to the permissive threshold used. These results are hypothesis-generating and require validation in future studies with larger cohorts. (See Methods Sect.  2.6 for details).

#### **miRNA-regulated networks**

**Fig. 3:**
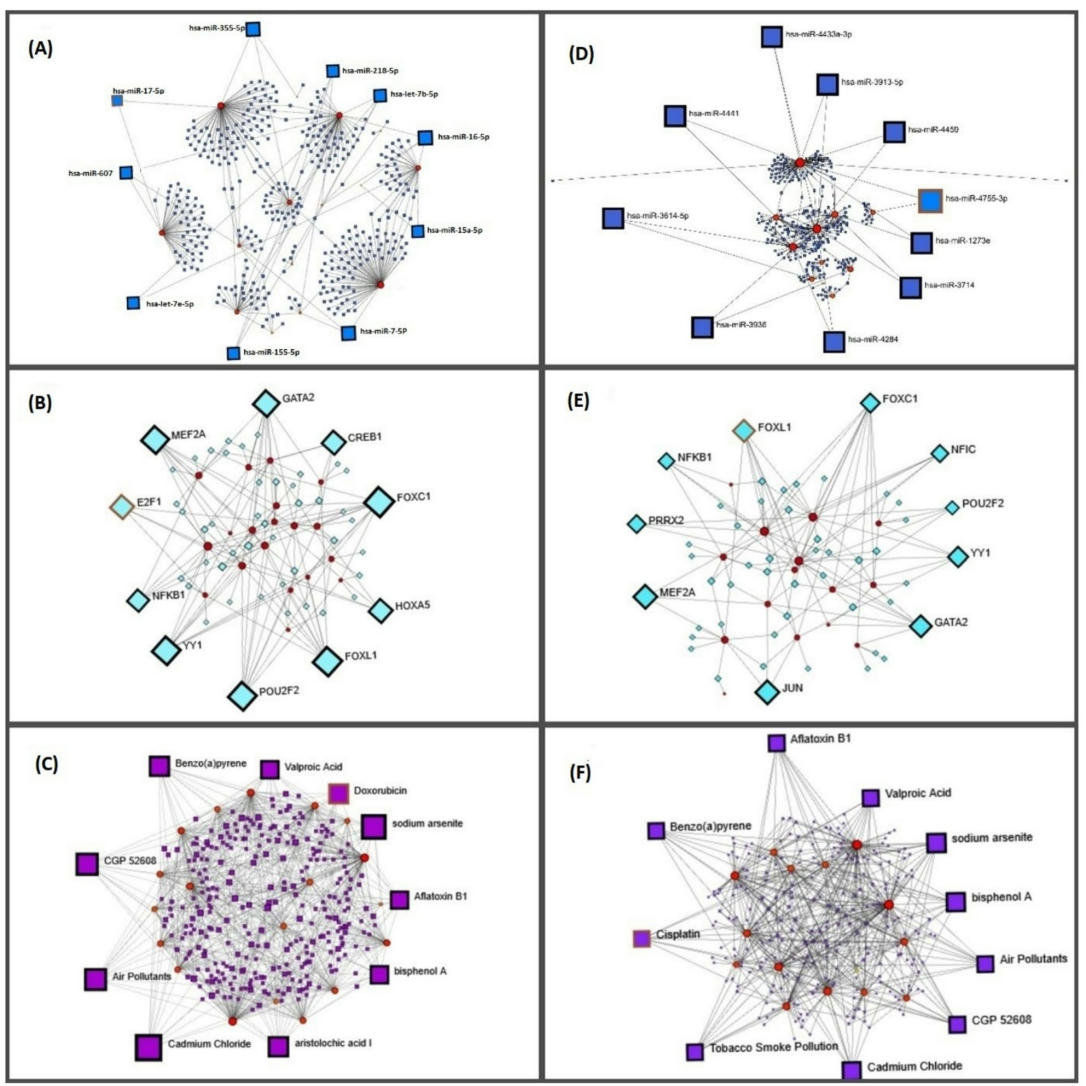
Sex-Specific Regulatory Networks for Pan-Brain Differentially Expressed Genes. Integrated regulatory network analysis of male and female consensus DEGs identifies key upstream regulators. (**A**, **D**) miRNA-mRNA interaction networks for (**A**) males and (**D**) females. Circular nodes represent upregulated genes; blue square nodes represent miRNAs. Hub miRNAs are those with the highest degree of connectivity. (**B**, **E**) Transcription factor (TF)-gene regulatory networks for (**B**) males and (**E**) females. Red circular nodes represent upregulated genes; light blue rhombus nodes denote TFs. The top 10 TFs, ranked by degree, are labeled. (**C**, **F**) Protein-chemical interaction networks for (**C**) males and (**F**) females. Red circular nodes represent significant genes; blue square nodes indicate chemical compounds or drugs. The top 10 interacting chemicals, based on degree, are shown. All networks were reconstructed and visualized using NetworkAnalyst, integrating data from JASPAR (TFs), miRTarBase (miRNAs; v9.0, experimentally validated interactions only), and the Comparative Toxicogenomics Database (CTD; chemicals)

Our miRNA analysis revealed distinct regulatory patterns between sexes that align with our functional enrichment findings. hsa-miR-16-5p, hsa-let-7b-5p, hsa-miR-607, hsa-miR-7-5p, hsa-miR-155-5p, hsa-miR-335-5p, hsa-miR-218-5p, hsa-miR-552-5p, hsa-miR-17-5p and hsa-let-7e-5p as the top ten microRNAs in men with bipolar disorder. The complete list of the top ten regulatory miRNAs in males included in Supplementary Table 6(S6) and shown in (Fig. 3A). hsa-miR-4768-3p, hsa-miR-21-5p, hsa-miR-8485, hsa-miR-3936, hsa-miR-3614-5p, hsa-miR-4755-3p, hsa-miR-769-3p, hsa-miR-4284, hsa-miR-6513-5p, hsa-miR-4270 as the microRNAs with the highest interaction with differentially expressed genes in women with bipolar disorder. The top ten regulatory miRNAs in females are included in Supplementary Table 7(S7) and shown in (Fig. 3D).

#### Transcription factor regulatory network analysis

Using upregulated genes from all studies, we identified regulatory elements derived from transcription factor (TF)-upregulated gene interactions. Red circular nodes represent upregulated genes, while light blue rhombus nodes denote TFs. Similarly, TF analysis revealed both shared and sex-specific regulatory programs. While TFs like YY1, GATA2, NFKB1 and FOXC1 emerged as central regulators in both sexes, suggesting common pathological mechanisms, there were also distinct sex-specific patterns.

In males, networks emphasized TFs involved in neurodevelopment and inflammation, such as MEF2A and NFKB1. The complete list of the top ten TFs in males included YY1, GATA2, FOXC1, CREB1, MEF2A, E2F1, POU2F2, NFKB1, HOXA5 and FOXL1 (Fig. 3B). In contrast, female-specific networks showed enrichment for TFs responsive to hormonal signals and involved in neurodevelopment, such as PRRX2. The top ten TFs in females were YY1, GATA2, FOXC1, NFIC, POU2F2, MEF2A, JUN, PRPX2, NFKB1 and FOXL1 (Fig. 3E) (Supplementary Tables 8, 9) (S8-S9). These findings provide mechanistic insights into how sex hormones and intrinsic biological differences might shape distinct molecular pathways in BD through upstream transcriptional control.

#### Protein-chemical interaction network

We constructed protein-chemical interaction networks for upregulated genes. In males, the network is presented in Fig. 3C, with blue square nodes indicating chemical compounds and red circles indicating significant genes. Top compounds in males included Bisphenol A, Valproic Acid, Sodium Arsenite, Benzo(a)pyrene, Aristolochic Acid I, Aflatoxin B1, CGP 52,608, Cadmium Chloride, Air Pollutants, Tretinoin and Doxorubicin. In females, top compounds included Sodium Arsenite, Bisphenol A, CGP 52,608, Valproic Acid, Aflatoxin B1, Air Pollutants, Tobacco Smoke Pollution, Cadmium Chloride, Cisplatin, Benzo(a)pyrene and Particulate Matter (Fig. 3F). (Supplementary Tables 10, 11) (S10-S11).

### Meta-analysis and functional enrichment in the striatal system

We performed a meta-analysis across all studies on 17,155 common genes that included GSE80336, GSE202537, and a subgroup of GSE80655 data sets that contain the striatal regions of the brain.

#### Meta-analysis in the striatal system

The striatal system showed more pronounced differences, with 289 significant genes (127 female-upregulated (LFC > 0), 162 male-upregulated (LFC < 0)) (FDR < 0.05). From these 289 genes, we found 42 genes (14.53%) with previously reported associations with bipolar disorder according to the Open Targets database (Table 3).

**Table 3:** Significantly impacted genes detected in the meta‑analysis of expression data from striatal system studies. Significantly impacted genes grouped by LFC value. Genes with a documented association with BD in the OpenTargets database are highlighted in bold

Direction of increment	Significant genes
LFC > 0	*AATK*,* ACAA1*,* ACSL4*, ***ACTRT2***, *ADAM33*,* ADAM9*, ***ADAMTS12***, *ADAMTS13*, ***ADAR***, *ADGRD2*,* ADGRE5*, ***ADNP***, *AGAP5*,* AKAP14*, ***AKTIP***, *ALKAL2*,* ANKLE2*,* ANKRD18CP*, ***ANKRD2***, *APBB2*,* ARFGAP2*,* ARHGEF4*,* ARHGEF5*,* ARHGEF6*,* ARID2*,* ARL13A*,* ARSG*, ***ASB16***, ***ATP2A2***, *ATP8B3*, ***ATRN***, ***BACE1***, *BBS10*,* BCAP29*,* BCAS3*, ***BCL11B***, *BFAR*,* BHLHE23*,* BPIFB2*,* BRCA1*, ***BRCA2***, *C10orf111*,* C19orf53*,* C19orf54*,* C19orf70*,* C1orf105*,* C2orf50*,* C2orf68*,* C2orf70*,* CA3*,* CABLES2*,* CABP5*,* CAND1*,* CATSPER1*,* CC2D2B*,* CCDC39*,* CCDC9B*,* CCL13*,* CD200*,* CD200R1*,* CD276*,* CDH12*, ***CDK11A***, ***CDK5RAP2***, ***CEACAM3***, *CHODL*,* CIB1*,* CIB2*,* CIC*,* CIDEA*,* CIR1*, ***CLN6***, *CLPTM1*,* CMC1*,* CMC4*,* CMKLR1*,* CMTM2*,* CMTM3*,* CMTM5*,* COPS2*,* CPNE8*,* CPNE9*,* CRIM1*, ***DAZAP1***, ***DCLK3***, *DEFB105B*,* DEFB108B*,* DEFB119*,* DGCR6L*,* DHDH*,* DIP2A*,* DLST*,* DNAJC8*,* DOLPP1*,* DPP7*,* E2F2*, ***EIF2B2***, *ELOVL7*,* ENSA*,* ERI3*,* FAF1*,* FAM182B*,* FAM183BP*,* FAM35DP*,* FAM3B*,* FAM47DP*,* FANCE*,* FBLL1*,* FBN2*,* FERMT3*,* FGD1*,* FGD2*,* FGF13*,* FIG4*,* FLT1*,* FLT3*,* FMNL1*,* FN3KRP*,* FOXRED2*,* FRMD5*,* FRMD7*,* KCTD3*,* LILRP2*,* LUC7L2*,* SLC9C2*,* SRGAP3*, ***CMPK1***
LFC < 0	*A1CF*,* A2ML1*,* AAR2*,* ABCB8*,* ABCC6*,* ABI1*,* ABLIM3*,* AC016026.1*,* AC091132.1*,* AC093323.1*,* AC123904.1*,* ACAD9*,* ACMSD*,* ACTL9*,* ACVR1B*,* ACY3*,* ADCK2*,* AFF2*,* AKIP1*,* ALDH18A1*,* ALDH6A1*,* ALDOA*, ***ALG9***, ***ALPK3***, *ALPP*,* AMN*,* AMN1*, ***ANO1***, *ANXA4*,* ANXA9*,* AP006437.1*, ***APPL2***, *AQP11*,* ARHGDIA*, ***ARL3***, *ARMCX3*,* ARRDC4*, ***ASXL3***, *ATP6AP2*,* ATXN7L1*,* BCAT2*,* BDH2*,* BEST1*,* BIN3*,* BLOC1S4*,* BOD1L1*,* BZW2*,* C11orf86*,* C15orf56*,* C16orf78*,* C19orf25*,* C1orf61*,* C21orf2*,* C22orf15*,* CA12*,* CABYR*,* CACNA1A*, ***CACNA1S***, *CADM3*,* CALHM1*,* CALN1*,* CALY*,* CAND2*, ***CBLN2***, *CBLN4*,* CBR4*,* CCDC25*,* CCDC58*, ***CCL20***, *CCNJ*,* CCSAP*,* CCZ1B*,* CDH18*,* CDH3*,* CDK12*,* CDKN3*,* CECR2*,* CEMIP*,* CEP41*,* CES2*,* CGREF1*, ***CHRNB4***, *CIAO1*,* CLC*,* CLEC17A*,* CLEC2D*,* CMA1*,* CMIP*,* CNFN*,* CNMD*, ***CNOT1***, *CNOT6*,* CNOT6L*,* COIL*,* COL9A2*,* COPS7A*,* COX6A2*,* COX6C*,* CRYBG3*,* CSRP3*,* CST8*,* CTF1*,* CTNND2*,* CTSW*,* CTU2*, ***CXCL11***, *CXCL17*,* CYP1A1*,* CYP27B1*,* CYP4B1*,* CYP4V2*, ***CYP7B1***, *DCBLD1*, ***DDC***, *DGLUCY*,* DKK1*,* DLC1*,* DLK2*, ***DNAH11***, *DRAP1*, ***DSCAM***, ***DSEL***, *DSG4*, ***DSP***, *DSPP*,* EAPP*,* ECH1*,* EEF1GP1*,* ELMO1*,* ELMOD2*,* EMC2*,* ENY2*,* EOMES*, ***EP300***, ***EPHX2***, *ERGIC1*,* ERICH3*,* ETAA1*,* EXTL3*,* FAM105A*,* FAM110C*,* FAM161A*,* FAM227A*,* FAM89B*,* FAM98C*,* FAM9B*,* FASTKD2*,* FBXL2*,* FBXL6*,* FBXO21*,* FCRL3*,* FLNC*,* FLT4*,* FOXL1*, ***GABRA6***, ***GABRG2***, *GALNT18*,* GAPDHP29*,* GBP5*,* GBP6*,* TTC14*,* ZNF263*

Key genes, grouped by their log2 fold change (LFC), with established BD associations (from OpenTargets) highlighted in bold

**Fig. 4:**
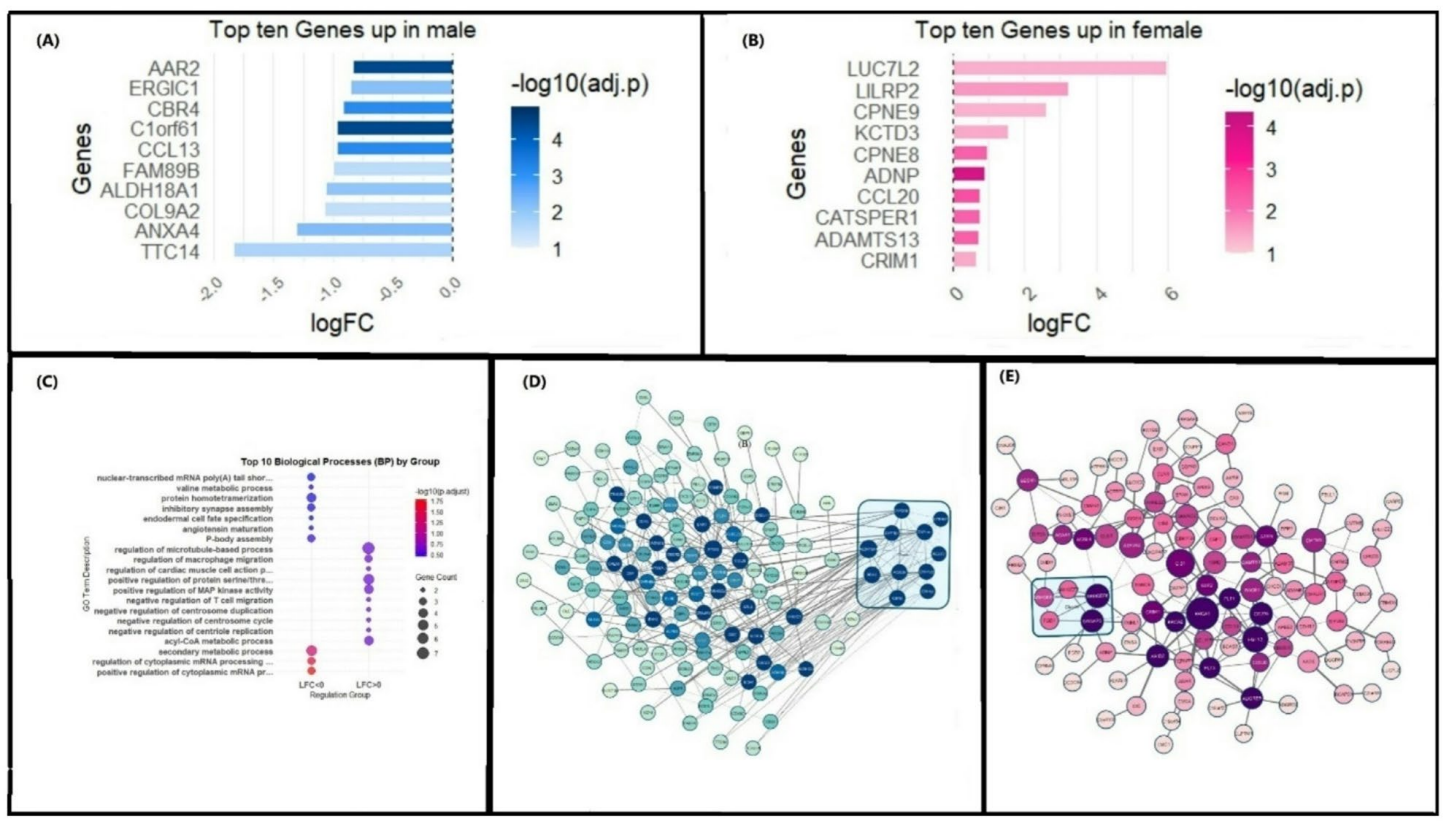
Sex-Biased Transcriptomic Signatures in the Striatal System. Sex-stratified meta-analysis of gene expression in striatal brain regions (dorsal striatum, nucleus accumbens, caudate, putamen). (**A**, **B**) The top ten significantly dysregulated genes in (**A**) males and (**B**) females, based on absolute log2 fold change (LFC). Error bars represent the 95% confidence interval. (**C**) Dot plot of significantly enriched Gene Ontology (GO) Biological Process terms for the striatal DEG sets. Dot size represents the gene ratio; color intensity represents the statistical significance (-log10(adjusted p-value)). (**D**, **E**) Protein-protein interaction (PPI) networks of striatal DEGs for (**D**) males and (**E**) females, showing the top functional module identified by MCODE clustering. Node color intensity corresponds to degree centrality; node size corresponds to betweenness centrality

In the striatum, male-upregulated genes were primarily associated with basic cellular and metabolic functions, such as mitochondrial enzymes (ALDH18A1) and proteins involved in cellular structure (COL9A2). Conversely, female-upregulated genes were linked to neuroprotective mechanisms (ADNP) and immune modulation (LILRP2), highlighting a sex-specific divergence in striatal pathology. The full list of the top ten upregulated genes in this region is available in Fig. 4(A, B). The full list of DEGs is available in Supplementary Table S12.

#### Over-represented functions and signaling pathways in the striatal system

ORA on all DEG in the striatum showed that enriched biological functions among male-upregulated genes (LFC < 0) were involved in RNA polymerase complexes (I/III) and nuclear transcriptional machinery, as well as endosomal and pre-synaptic elements. By contrast, female upregulated genes (LFC > 0) were enriched for postsynaptic structures (dendritic spines, PSD) and cytoskeletal motility components (lamellipodia, filopodia). Simultaneous upregulation of both focal adhesions and cell-substrate contacts, and as motility structures, was also detected. Nuclear envelope downregulation opposed distal axon upregulation, illustrating the compartmentalized alterations in neuronal structure (Fig. 4C).

Furthermore, to detect significant pathways (adjusted p-value < 0.05) specific to male and females across all upregulated genes in males and females, we used a variety of pathway databases (KEGG, WikiPathways, Reactome, BioPlanet, Elsevier and BioCarta). Oxidation by Cytochrome P450 WP43 was the most significant metabolic pathway in male and CDC42 GTPase Cycle and *BRCA1*-dependent Ub-ligase activity Homo sapiens h bard1Pathway and Cell Death Signaling via NRAGE, NRIF, and NADE were the most significant pathways in female (Supplementary Tables 13,14) (S13-S14).

#### Protein–protein interaction networks Hub Gene Identification in the striatal system

The top cluster identified from the PPI network of males and females in striatal studies is shown in Fig. 4 (D, E). The consensus hub gene analysis for the striatal system revealed distinct key regulators for each sex. In males, the hub genes were *BDH2* and *CYP1A1*. In females, the consensus hub genes were *CD276*, *BRCA1*, and *FGF13*. (supplementary File15-16) (S15-S16). (See Methods Sect.  2.6 for details).

#### miRNA-regulated networks

**Fig. 5:**
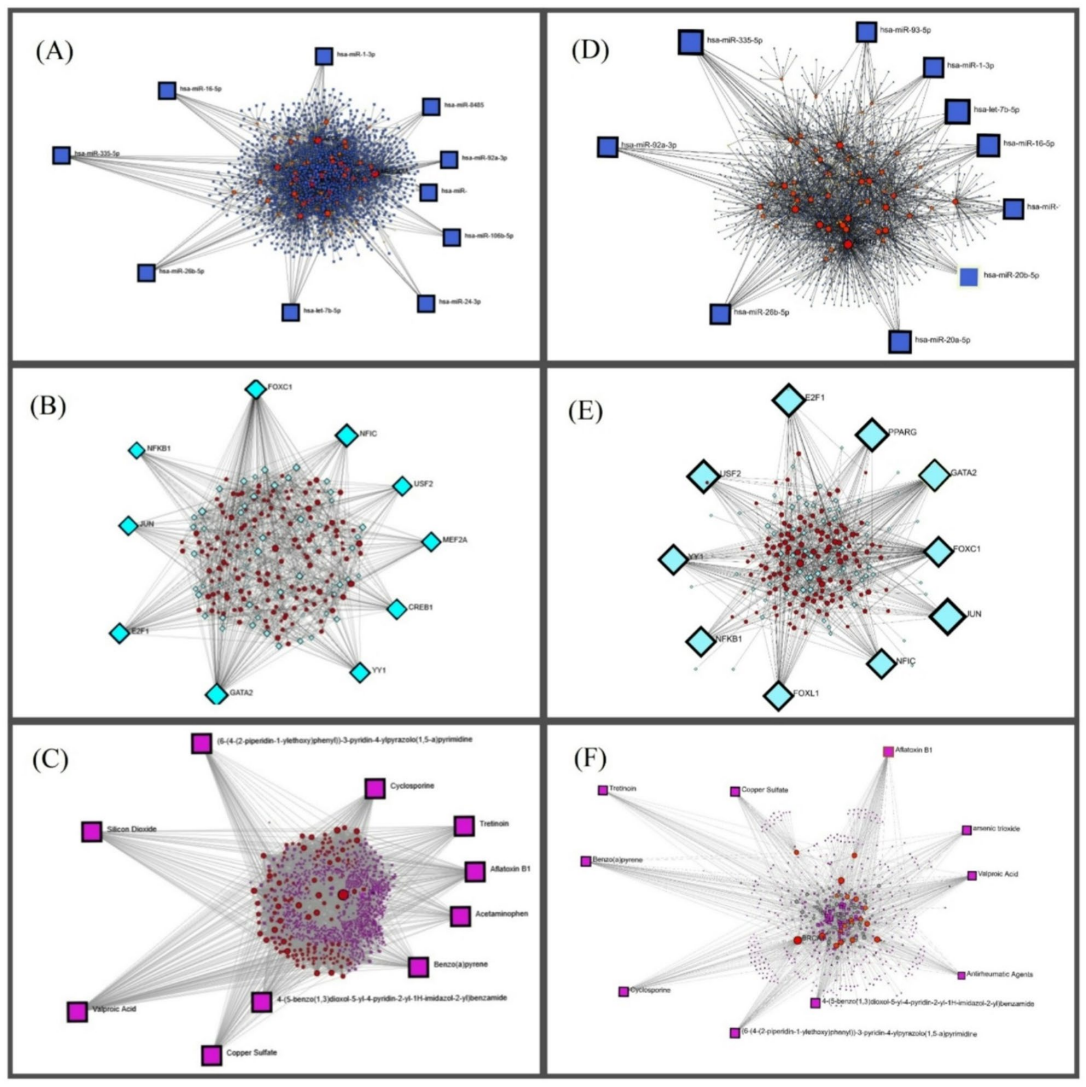
Sex-Specific Regulatory Networks for Striatal Differentially Expressed Genes. Regulatory network analysis of sex-specific DEGs from the striatal meta-analysis. (**A**, **D**) miRNA-mRNA interaction networks for (**A**) males and (**D**) females. (**B**, **E**) Transcription factor (TF)-gene regulatory networks for (**B**) males and (**E**) females. (**C**, **F**) Protein-chemical interaction networks for (**C**) males and (**F**) females

In males, the top ten regulatory miRNAs including hsa-mir-355-5p, hsa-mir-16-5p, hsa-let-7b-5p, hsa-mir-20a-5p, and hsa-mir-26b-5p, hsa-mir-7b-5p, hsa-mir-24-3p, hsa-mir-106-5p, hsa-mir-92a-3p, hsa-mir-1-3p, and hsa-mir8485 (Fig. 5A). In females, the top ten key miRNAs: hsa-mir-20a-5p, hsa-mir-26b-5p, hsa-mir-92a-3p, hsa-mir-355-5p, hsa-mir-16-5p, hsa-mir-93-5p, hsa-mir-17-5p, hsa-mir-1-3p, hsa-mir-7b-5p and hsa-mir-20b-5p (Fig. 5D) (Supplementary Tables 17,18)(S17,S18).

#### Transcription factor regulatory network analysis

Our analysis of striatal studies identified sex-specific regulatory networks through transcription factor (TF) interactions with upregulated genes. The network visualization represents the most significant upregulated genes as red circular nodes and TFs as blue square nodes based on degree. The top TFs in males were PPARG, USF2, NFKB1, FOXL1, NFIC, JUN, YY1, GATA2, FOXC1 and E2F1 (Fig. 5B), while in females, top TFs were YY1, GATA2, FOXC1, NFIC, CREB1, MEF2A, NFKB1, USF2, JUN, and FOXL1 (Fig. 5E). (Supplementary Tables 19,20) (S19-S20).

#### Protein-chemical interaction network

We constructed protein-chemical interaction networks for upregulated genes. In males (Fig. 5C), top compounds included valproic acid, copper sulfate, aflatoxin B1, benzo(a)pyrene, cyclosporine, 4-(5-benzo [1,3] dioxol-5-yl-4-pyridin-2-yl-1 H-imidazol-2-yl) benzamide, silicon dioxide, acetaminophen, and (6-(4-(2-piperidin-1-ylethoxy) phenyl))-3-pyridin-4-ylpyrazolo(1,5-a) pyrimidine. In females (Fig. 5F), top compounds included valproic acid, benzo(a)pyrene, aflatoxin B1, cyclosporine, 4-(5-benzo[1,3]dioxol-5-yl-4-pyridin-2-yl-1 H-imidazol-2-yl)benzamide, antirheumatic agents, arsenic trioxide, tretinoin, (6-(4-(2-piperidin-1-ylethoxy)phenyl))-3-pyridin-4-ylpyrazolo(1,5-a)pyrimidine, and copper sulfate.(Supplementary Tables 21,22) (S21-S22).

### Meta-analysis and functional enrichment in the limbic system

Meta-analysis on 5571 common genes in the GSE42546 dataset and a subset of GSE80655 that included limbic brain regions showed that there were only two genes that were upregulated in women, namely *SRSF4* (serine/arginine-rich splicing factor 4 involved in mRNA splicing regulation) and *PPME1* (protein phosphatase methyl esterase 1 involved in demethylation and modulation of protein phosphatase 2 A activity). Full meta-analysis results for these two DEGs of the limbic system, including Log2FC, 95% confidence intervals, and heterogeneity statistics (I²), are provided in Supplementary Table 23(S23).

**Fig. 6:**
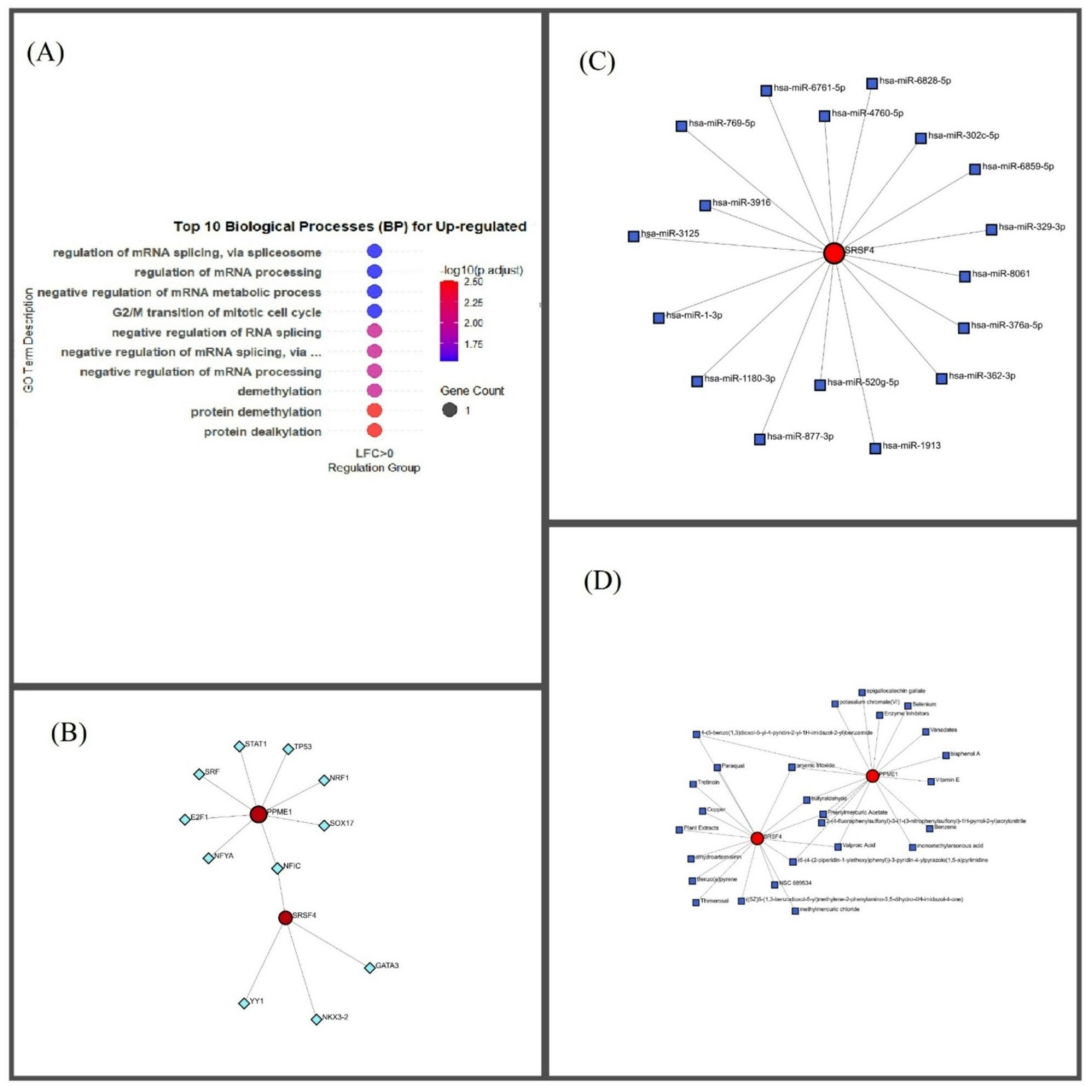
Functional Enrichment and Regulatory Landscape of the Limbic System. (**A**) Dot plot of significantly enriched Gene Ontology (GO) Biological Process terms for the two female-upregulated genes (SRSF4, PPME1) identified in the limbic meta-analysis. (**B**) Transcription factor (TF)-gene interactions for the significant limbic DEGs. (**C**) miRNA-mRNA interaction network. (**D**) Protein-chemical interaction network.

The top biological processes for these genes are described in Fig. 6A. TF interactions, miRNA interactions, and protein-chemical interactions in the female limbic system are shown in Fig. 6(B, C, and D) (Supplementary Tables 24,25,26) (S24-S25-S26).

### Assessment of age confounding in sex-specific signals

We also conducted a meta-regression model to explore the confounding role of age difference between the cases and control groups. This analysis was performed on two levels: the all-brain analysis and the striatal region analysis.

Remarkably, none of the sex-biased genes identified reached significance for age-dependent effects in the meta-analyses in all studies and the striatal system (age_padj > 0.05 for all DEGs). (supplementary Tables 27,28) (S27-S28). This suggests that the patterns of sexual dimorphism reported here are consistent and not influenced by age disparity across the studies.

In contrast, because of a limited number of studies regarding the limbic system, meta-regression was not possible for this region. We can thus not draw any conclusions as to the presence or absence of an age confounder effect in this system.

### Overlap between disease effects and baseline sex differences

To address whether our observed sex-specific disease effects were built upon inherent biological differences between sexes, we first identified genes with significant baseline sex differences in healthy controls. Our meta-analysis revealed 45 genes that were significantly differentially expressed between healthy males and females (FDR < 0.05) (Supplementary Table S29). We then compared this baseline list with our sex-specific disease gene list. This comparison revealed a striking pattern: there was no overlap between the baseline sex-difference genes and either the 34 genes identified in our pan-brain analysis or the 289 striatal genes.

### Quantitative assessment of sex-specific interaction effects

To complement our primary meta-analysis and directly quantify the divergence of transcriptional responses between sexes, we performed a quantitative interaction analysis. First, we assessed the overall correlation between disease effect sizes (log2FC) in females and males across all genes common to the four studies. This analysis revealed a statistically significant but negligible correlation (Pearson *r* = -0.122, *p* = 2 e-16), indicating that the magnitude and direction of gene expression changes in BD are largely independent between sexes (Supplementary Figure S30). Second, we performed a formal meta-analysis to test for significant sex-by-diagnosis interaction effects for each gene. The distribution of the interaction term (β_female - β_male) was bimodal, confirming that for a substantial subset of genes, the disease effect operates in opposite directions depending on sex (Supplementary Figure S31). Notably one gene, MEF2C, emerged with strong and significant sex-interaction effects (FDR < 0.05). MEF2C exhibited a trend towards downregulation in females (β ≈ β = -0.364) but upregulation in males (β ≈+ 0.244). (Supplementary Table S32). This gene, with opposing effect directions, exemplify the qualitative divergence in molecular pathology between men and women with BD.

## Discussion

Our integrated meta-analysis provides, to our knowledge, the first systematic evidence for distinct differences between male and female BD patients across brain regions. The most prominent sex-specific transcription dynamics emerged in the striatum, a brain region closely linked to motivation and reward, with modest commonalities between sexes. These patterns defined unique sets of biomarkers and pathways that correspond with established neurobiological principles. This conclusion of biological divergence is strongly reinforced by our supplementary quantitative interaction analysis, which found a negligible correlation (*r* ≈ -0.122) between disease effect sizes in females and males. Furthermore, we identified just one gene, MEF2C, that displayed opposing pattern of dysregulation, downregulation in females versus upregulation in males, exemplifying a qualitative, sex-dependent molecular response to the illness.

Further examination of hub genes in four independent centrality measurement schemes indicated sex- and region-specific molecular epicenters. In females, the hub genes *PHF20L1* and *PANK2* emerged as central nodes. *PHF20L1* is involved in epigenetic regulation and proteostasis, while *PANK2* plays a role in mitochondrial energy metabolism. Dysregulation of these pathways aligns with the female-specific themes of stress response and synaptic plasticity observed in our study, suggesting that disruptions in epigenetic programming and mitochondrial energetics may contribute to mood instability in women with BD. In males, *PPIG* (Peptidylprolyl Isomerase G) was identified as a key hub gene. Known for its role in RNA processing and protein folding, PPIG functions as a nuclear cyclophilin that regulates alternative splicing. Its identification suggests a male-specific pathophysiology characterized by critical disruptions in nuclear RNA metabolism and splicing, processes essential for neuronal function. Together, these sex-specific hub genes support the conclusion that BD involves fundamentally different molecular architectures in males and females. It is important to emphasize that the identification of PPIG in males and PHF20L1 and PANK2 in females as hub genes in the pan-brain analysis should be considered exploratory due to the limited gene set available after aggregation and the permissive interaction threshold applied. These findings warrant cautious interpretation and require validation in larger, independent cohorts to confirm their role as central nodes in the sex-specific molecular pathology of bipolar disorder.

The most pronounced sex-specific signatures were evident in the striatal system meta-analysis. This sex-specific hub organization was further amplified in the striatum, with females highlighting *CD276* (immune function), *BRCA1* (DNA repair and neurite remodeling), and *FGF13* (neuronal excitability), while males strongly centered on *BDH2* (ketone metabolism and redox balance) and *CYP1A1* (xenobiotic metabolism).

Sex-specific molecular dysfunctions were also identified in the pan-brain meta-analysis. In females, the *FSHR*, *POF1B* and PCSK2 emerged as key differentially expressed genes. While FSHR is classically known for its role in ovarian function and FSH signaling, it is also expressed in the brain, particularly in the hippocampus, and has been directly implicated in mood regulation, as Fshr-knockout mice exhibit affective disorder-like behaviors [40, 56]. POF1B, involved in cytoskeletal organization, may contribute to synaptic structure and plasticity [40]. PCSK2, a prohormone convertase, processes multiple neuropeptides in the brain, including BDNF, CRH, and neurotensin, that are important for stress response and mood regulation, extending its relevance beyond insulin metabolism [41]. In males, notable brain-related genes included RYR1, a ryanodine receptor implicated in neuronal calcium homeostasis and pathways relevant to antidepressant response [42]; RIMS2, which is involved in the regulation of synaptic vesicle exocytosis and synaptic plasticity [43]; and NCOR1, a transcriptional regulator in epigenetic processes and neuronal stress responses [44]. Together, these sex-specific gene sets indicate divergent pathophysiological pathways: neuropeptide processing and cytoskeletal plasticity in females, versus calcium signaling, synaptic release machinery, and transcriptional regulation in males.

The biological relevance of our results is supported by a high degree of overlap (10 out of 34 genes) with well-established BD-associated genes in the Open Targets database.

Our study moves the field forward by revealing unique sex-specific gene networks through meta-analysis, moving beyond correlation to define key transcriptional drivers.

In the striatum, male-specific expression involved cytochrome P450 genes (e.g., *CYP1A1*, *CYP27B1*) and mitochondrial metabolism genes (e.g., *BDH2*,* ALDH18A1*), which are implicated in oxidative metabolism, energy homeostasis, and neurotransmission [45]. Their dysregulation is associated with impulsivity, substance abuse [46], and mitochondrial dysfunction [47, 48]. Conversely, females showed upregulated expression of Rho GTPase regulators (e.g., *ARHGEF4*,* SRGAP3*), *BRCA1*, and *FGF13*, all indicating increased cytoskeletal dynamics [49, 50], which may represent resilience pathways [51] to mediate synaptic plasticity in striatal circuits [52].

These striatal sex differences may undergird male-biased metabolic vulnerabilities and female-biased susceptibility to depression episodes and disrupted dopamine signaling [53, 54]. Notably, these bulk-tissue-based signatures may originate from shifts in cellular composition or cell-type-specific expression, a critical limitation that necessitates validation using cell-type-resolving approaches.

Although the *PHF20L1* and *PANK2* genes have not been directly implicated in genetic studies of bipolar disorder, there is evidence of their indirect association with a broader range of mood and neuropsychiatric disorders. It has shown an association of the PHF20 gene (PHF20L1 family) with bipolar disorder and depression, as well as related traits such as Neuroticism. Also, a clinical report of a bipolar disorder patient with severe basal ganglia atrophy, although not carrying a *PANK2* mutation, suggests a role for this gene in neurodegeneration associated with iron accumulation (NBIA) and its possible association with neural circuits involved in bipolar disorder [55]. Therefore, the identification of these two genes as female-specific hubs in our study could open a new window towards understanding the molecular mechanisms involved in the gender dimorphism of bipolar disorder.

Our analysis also highlights *FSHR* [56], and *RPS6KA2* [57] as crucial female-specific genes and supports previous evidence for neurotrophic signaling dysregulation in BD. These findings align with a broader framework in psychiatric research; for instance, our earlier works observed reduced GDNF (another neurotrophic factor) in schizophrenia [58] and Major depressive disorder [59], and Bipolar disorder [60] suggesting imbalances in neurotrophic pathways may be a transdiagnostic mechanism in severe mental illnesses.

One of the notable genes upregulated in females in the pan-brain analysis is PACS1 (Phosphofurin Acidic Cluster Sorting Protein 1). This gene has been implicated as a direct functional target in genetic studies of bipolar disorder. Research published in Genome Medicine (2022) [61] utilizing a functional genomics approach demonstrated that functional single nucleotide polymorphisms (SNPs) within BD-associated risk loci can influence the expression of PACS1 in the brain. More importantly, overexpression of this gene in primary mouse cortical neurons led to a reduction in dendritic spine density. This provides a potential mechanistic link to BD pathogenesis, as alterations in synaptic plasticity and dendritic morphology are recognized as hallmarks of neuropsychiatric disorders. The observed upregulation of PACS1 specifically in female patients with BD in our study aligns with these findings and suggests that disruption of synaptic flexibility may represent a sex-specific pathogenic mechanism.

The inclusion of PDLIM5 in our list of male-upregulated genes is notable, as previous gene expression studies have identified PDLIM5 as being associated with bipolar disorder. Although those earlier findings were not sex-specific, they established a role for this gene in the pathophysiology of BD. Given that PDLIM5 is involved in neuronal calcium signaling and synaptic function, processes central to mood disorders, our observation of its specific upregulation in males may point toward a sex-differential mechanism. This finding warrants further investigation into sex-specific expression patterns of *PDLIM5*.

The distinct gene expression profiles between male and female patients point toward divergent biological pathways underlying bipolar disorder (BD). In women, upregulated genes in the pan-brain are involved in synaptic plasticity (*PACS1*) [61], MAPK signaling (*RPS6KA2*) [62], hormonal regulation (FSHR) [63], and inflammation (NLRC4) [64]. This suggests that the pathophysiology of BD in females may be more closely associated with neuroendocrine-immune interactions. While our study cannot establish causality, this neuroendocrine-immune axis could contribute to the higher rates of rapid cycling, depressive episodes, and comorbid autoimmune conditions observed in women with BD.

In contrast, genes upregulated in the pan-brain analysis in men are enriched for synaptic vesicle release (RIMS2) [43], calcium homeostasis (RYR1) [65], and synaptic function (PDLIM5) [66], along with nuclear receptor signaling (NCOR1)(67). These processes are fundamental to neurotransmitter release and neuronal excitability.

While Perez et al. (GSE202537) [68] first reported this difference between two sex for immune and angiogenesis pathways, our meta-analysis generalizes it on a larger scale and identifies distinct biological pathways specific to each sex. In females, upregulated genes were predominantly involved in synaptic plasticity, MAPK signaling, neuroinflammation, and hormonal regulation. In males, they were enriched for synaptic vesicle release, calcium homeostasis, and nuclear receptor signaling. This concordance between independent studies provides strong evidence for fundamental molecular differences between sexes in mental illness and confirms the need for sex-specific research and treatment strategies.

Our research reveals a multi-layered regulatory architecture in bipolar disorder, with both universal and sex-specific components. At the core of this network, a set of transcription factors (YY1, GATA2, FOXC1, *MEF2A*, and FOXL1) emerged as fundamental regulators in both sexes, making them promising universal therapeutic targets. Beyond this shared core, we observed distinct sex-specific TF enrichment. Male-specific networks was enriched for *HOXA5*, involved in neuronal differentiation [69] and brain development [70], suggesting male-biased neurodevelopmental trajectories in psychiatric disorders. In females, *PRRX2* as female-specific emerged as a key regulator, a TF known to be responsive to hormonal fluctuations [20] and involvement in female-specific transcriptional networks [17]. These findings highlight how upstream regulatory control may differ between sexes in BD.

In males, several miRNAs with established roles in neuropsychiatric disorders emerged as key regulators. hsa-miR-155-5p is a key pro-inflammatory miRNA linked to immune dysregulation in bipolar disorder [71], while hsa-miR-16-5p regulates serotonin transporter expression and stress response [72]. miR-17-5p, also identified in males, has been implicated in depression-related signaling pathways [73], and miR-7-5p, associated with schizophrenia pathology [74]. In contrast, the top miRNAs in females included several that have been less studied in psychiatric disorders (e.g., hsa-miR-4768-3p, hsa-miR-3936, hsa-miR-3614-5p), alongside more established miRNAs like hsa-miR-21-5p. This mix of well-characterized and potentially novel regulators suggests that female BD may involve both known and under-explored post-transcriptional mechanisms.

The translational implications of these findings are underscored by our analysis of protein-chemical interactions. While some compounds, like valproic acid and aflatoxin B1, target common pathways, we also uncovered a network of sex-specific interactions. This implies that an individual’s risk and symptom profile may be shaped by environmental and pharmacological exposures in a sex-dependent manner. Collectively, these interconnected findings, from transcription factors to miRNAs to chemical interactions, provide a compelling, multi-level argument for the necessity of sex-specific research to develop more effective, personalized treatments and safety guidelines for bipolar disorder.

Our analysis of the limbic system revealed minimal gene expression changes, with only two genes upregulated in females. This null result is likely due to insufficient statistical power, as fewer studies focused on limbic regions were available. The stark contrast with the pronounced alterations observed in the striatum underscores the regional specificity of sex-biased transcriptomic signatures in BD.

Our comparative analysis of sex-specific genes in disease and baseline sex differences revealed a complete lack of any overlap between the 34 pan-brain genes or the 289 striatal system brain regions with the 45 genes with significant expression differences between healthy females and males. This finding has important biological implications. First, it suggests that the transcriptional changes observed in females with bipolar disorder are not simply an exacerbation or amplification of pre-existing gender dimorphisms. Rather, these changes represent new pathological signatures that emerge specifically in the disease state. Second, the complete independence of these gene sets suggests that bipolar disorder activates distinct molecular pathways in women that are not related to normal sex-specific biology. This provides strong evidence that the pathophysiology of bipolar disorder in women involves unique mechanisms that cannot be predicted or explained by studying baseline sex differences alone. Third, from a therapeutic perspective, these findings highlight the need to develop It reinforces sex-specific treatment strategies, because the molecular targets involved in female disease are fundamentally different from male disease and healthy gender differences. The lack of overlap also increases confidence that the female-specific genes we identified are truly disease-associated and not confounded by inherent biological variation between the sexes. This model, in which disease-associated genes are distinct from basic sex differences, underpins the notion that bipolar disorder is not simply “built” on natural sex biology, but rather creates sex-specific pathological states that require dedicated research and targeted interventions.

### Limitations

While our study provides important insights, several limitations must be acknowledged. The most significant issue is a pronounced male bias in the available brain tissue samples (61% male, 106/173). This imbalance provided greater statistical power to detect effects in males. Therefore, the larger number of differentially expressed genes (DEGs) identified in males should not be interpreted as evidence of greater biological magnitude, but rather as a reflection of this power differential. While our sex-stratified analytical design minimizes direct comparison bias, the greater power in the male cohort means our findings in females are likely conservative, representing only the most robust effects. The female-specific findings, while robust and statistically significant, likely represent only the most pronounced effects, and more subtle female-specific signatures may have been undetected. Future studies with balanced cohorts are needed to identify the full spectrum of female-specific molecular signatures.

Furthermore, as with all post-mortem brain research, our analysis was confronted with inevitable confounders. We lacked detailed medication histories for most donors, and since psychotropic drugs profoundly alter gene expression, our results likely reflect a blend of the disease’s true biology and adaptations to treatment. We also had limited ability to account for key biological variables like post-mortem interval or RNA integrity, as this data was insufficiently reported across the original studies.

A more fundamental constraint is inherent to the method we used: analyzing bulk tissue. This approach prevents us from discerning whether the genetic changes we observed are due to alterations in cellular composition (e.g., glial vs. neuronal counts) or to changes in gene activity within specific cell populations. Clarifying this will require future studies using single-cell or spatial transcriptomics.

Furthermore, the integration of datasets using different tissue sampling techniques (e.g., laser-captured neurons from one study with bulk tissue from others) introduces an additional layer of heterogeneity, which, while accounted for by our random-effects model, complicates the direct interpretation of cell-type-specific signals.

Finally, our strategy of analyzing three brain regions and seeking a consensus, while methodically sound, increases the risk of missing subtle, region-specific effects and may be overly conservative. This is probably why we saw such limited changes in the limbic system. Therefore, our key observations require confirmation in larger, more balanced cohorts and with higher-resolution techniques.

## Conclusion

This study reveals that bipolar disorder engages largely distinct biological mechanisms in men and women. In the pan-brain analysis, we identified 34 sex-specific genes, with females showing dysregulation in hormonal signaling (*FSHR*), synaptic plasticity (PACS1), and inflammatory pathways (*NLRC4*), while males exhibited alterations in calcium homeostasis (*RYR1*), synaptic vesicle release (*RIMS2*), and transcriptional regulation (*NCOR1*). The most significant differences were found in the striatal system, where men showed disruptions in mitochondrial metabolism (BDH2) and xenobiotic processing (CYP1A1), while women exhibited changes in immune function (CD276), DNA repair (BRCA1), and neuronal excitability (FGF13). The negligible correlation between male and female effect sizes (*r* = -0.122) and the identification of MEF2C as a gene with opposing dysregulation between sexes further confirm that the molecular pathology of BD is qualitatively different in men and women. Moreover, the complete lack of overlap between disease-associated genes and genes showing baseline sex differences strongly suggests that these sex-specific signatures are true pathological features rather than amplifications of normal sexual dimorphism. These findings demonstrate that we cannot fully understand this disorder without considering sex-specific pathways. Future research and treatments must account for these fundamental differences to develop truly effective, personalized approaches for bipolar disorder.

## Supplementary Information

Below is the link to the electronic supplementary material.

Supplementary Material 1

Supplementary Material 2

Supplementary Material 3

Supplementary Material 4

Supplementary Material 5

Supplementary Material 6

Supplementary Material 7

Supplementary Material 8

Supplementary Material 9

Supplementary Material 10

Supplementary Material 11

Supplementary Material 12

Supplementary Material 13

Supplementary Material 14

Supplementary Material 15

Supplementary Material 16

Supplementary Material 17

Supplementary Material 18

Supplementary Material 19

Supplementary Material 20

Supplementary Material 21

Supplementary Material 22

Supplementary Material 23

Supplementary Material 24

Supplementary Material 25

Supplementary Material 26

Supplementary Material 27

Supplementary Material 28

Supplementary Material 29

Supplementary Material 30

## Data Availability

The data used for the analyses described in this work are publicly available atGEO (24)The accession numbers of the GEO datasets downloaded are GSE80336, GSE80655, GSE202537, GSE42546.

## Code availability

The code developed for the analyses described in this work has been made. publicly available at GitHub( https://github.com/kazemisafa94-boop/Bipolar-meta-analysis/blob/3740010a5de1f6739f4f7f3499859002a19b69ca/scripts%20codes). All software and versions used are detailed in Additional file 2: Table S2.

## Abbreviations

BD: Bipolar disorder
DEGs: Differentially expressed genes
LFC: Log2 fold change
FDR: False discovery rate
PPI: Protein-protein interaction
GRN: Gene regulatory network
TFs: Transcription factors
miRNA: MicroRNA
ORA: Over-representation analysis
PCA: Principal component analysis
REML: Restricted maximum likelihood
SE: Standard error
CI: Confidence interval
GEO: Gene expression omnibus
HGNC: HUGO gene nomenclature committee
CTD: Comparative toxicogenomics database
KEGG: Kyoto encyclopedia of genes and genomes
NAc: Nucleus accumbens
DG: Dentate gyrus
PFC: Prefrontal cortex
DLPFC: Dorsolateral prefrontal cortex
ACC: Anterior cingulate cortex
PRISMA: Preferred reporting items for systematic reviews and meta-analyses
