# Supplementary material for: Sex-biased transcriptomic landscapes in bipolar disorder: integrating neurobiology and clinical heterogeneity through cross-study meta-analysis
Source: Biol Sex Differ. 2026 May 8;17:125. doi: 10.1186/s13293-026-00870-4 (PMC13321544; doi:10.1186/s13293-026-00870-4)
Supplement: Supplementary file 1 — Supplementary Material 1 [file 13293_2026_870_MOESM1_ESM.docx]

**Supplementary Table 1:R software Packages were used in this research**

| **Package Name** | **Version** | **Purpose** |
| --- | --- | --- |
| **DESeq2** | 1.46.0 | Differential expression analysis to identify sex-stratified DEGs. |
| **limma** | 3.62.2 | Batch effect correction using the removeBatchEffect() function. |
| **biomaRt** | 2.62.1 | Gene annotation; converting raw gene identifiers to official HGNC symbols. |
| **metafor** | 4.8-0 | Cross-study meta-analysis using a random-effects model (REML). |
| **clusterProfiler** | 4.14.6 | Functional enrichment analysis (Over-Representation Analysis - ORA) of Gene Ontology terms. |
| **VennDiagram** | 1.7.3 | Identifying overlapping DEGs across datasets and for hub gene consensus. |
| **ggplot2** | 3.5.1 | Generation of all publication-quality figures and plots (e.g., bar charts, dot plots). |
| **RColorBrewer** | 1.1-3 | Providing color palettes for figures. |
| **reshape2** | 1.4.4 | Data wrangling and transformation for analysis and visualization. |
| **dplyr** | 1.1.4 | Data manipulation, filtering, and management. |
| **stringr** | 1.5.1 | String manipulation and handling of gene names. |
| **readr** | 2.1.5 | Efficient reading of large data files. |
| **tidyr** | 1.3.1 | Data tidying and organization. |
| **pheatmap** | 1.0.12 | Creating heatmaps to visualize gene expression patterns |
