# Supplementary material for: Sex-biased transcriptomic landscapes in bipolar disorder: integrating neurobiology and clinical heterogeneity through cross-study meta-analysis
Source: Biol Sex Differ. 2026 May 8;17:125. doi: 10.1186/s13293-026-00870-4 (PMC13321544; doi:10.1186/s13293-026-00870-4)
Supplement: Supplementary file 4 — Supplementary Material 4 [file 13293_2026_870_MOESM4_ESM.docx]

**Table5.** Significant signaling pathway in female upregulated genes in All studies

| Category | Pathway | Adjusted p-  value | Genes in the  pathway |
| --- | --- | --- | --- |
| **Biocarta**  **2016** | Regulation of Spermatogenesis by CREM Homo sapiens h cremPathway | 0.007 | FSHR |
| **Elsevier** | Proteins Involved in Primary Ovarian Insufficiency | 0.039568510702488055 | POF1B;FSHR |
|  | Testicular AMH Production | 0.039568510702488055 | FSHR |
|  | FOXO3 and PTEN Inactivation in Premature Ovarian Failure (Mouse Model) | 0.039568510702488055 | FSHR |
|  | Genes with Mutations Associated with Primary Ovarian Insufficiency | 0.039568510702488055 | FSHR |
|  | Sister Chromatid Cohesion | 0.039568510702488055 | NIPBL |
|  | GCG and PPY Regulate Metabolism and Satiety | 0.039568510702488055 | PCSK2 |
|  | POMC Secretion in adenohypophysis | 0.039568510702488055 | PCSK2 |
|  | Thyrotropin Releasing Hormone (TRH) Hypothalamic Release in non-Autoimmune Hypothyroidism | 0.039568510702488055 | PCSK2 |
|  | alpha-Cell to beta-Cell Interconversion (Hypothesis) | 0.039568510702488055 | PCSK2 |
|  | FSH Action in Polycystic Ovary Syndrome | 0.039568510702488055 | FSHR |
|  | ECL-cell: CHGA and Histamine Synthesis | 0.039568510702488055 | PCSK2 |
|  | G-cell: Gastrin Secretion | 0.039568510702488055 | PCSK2 |
|  | LH and FSH Signaling Impairment in Primary Ovarian Insufficiency | 0.039568510702488055 | FSHR |
|  | LH and FSH Signaling and Steroidogenesis | 0.039568510702488055 | FSHR |
|  | L-cell: GCG, PYY and 5-HT Release | 0.039568510702488055 | PCSK2 |
|  | Leptin and CCK8 Activate Nodose Ganglia Neuron | 0.039568510702488055 | PCSK2 |
|  | Central Hypothyroidism | 0.039568510702488055 | PCSK2 |
|  | Tertiary Hypothyroidism | 0.039568510702488055 | PCSK2 |
|  | Sertoli Cells Dysfunction Causes Male Infertility | 0.039568510702488055 | FSHR |
|  | Ovulation Block | 0.039568510702488055 | FSHR |
|  | NOD-like Receptors | 0.039568510702488055 | NLRC4 |
|  | Insulin Synthesis in beta-Cell | 0.04010255753388379 | PCSK2 |
|  | Blau Syndrome | 0.040634528870567295 | NLRC4 |
|  | Proteins Involved in Azoospermia | 0.040634528870567295 | FSHR |
|  | Golgi to Endosome Transport | 0.040634528870567295 | PACS1 |
|  | Steroidogenesis Impairment in Polycystic Ovary Syndrome | 0.040634528870567295 | FSHR |
|  | Ras-GRF Regulation Signaling | 0.040634528870567295 | RPS6KA2 |
|  | MC1R in anti-Inflammatory Signaling | 0.040634528870567295 | PCSK2 |
|  | Polycystic Ovary Syndrome | 0.046237524975354334 | FSHR |
|  | *Proteins Involved in Female Infertility* | *0.039568510702488055* | *POF1B;FSHR* |

| *Category* | *Pathway* | *Adjusted p-*  *value* | *Genes in the*  *pathway* |
| --- | --- | --- | --- |
| ***Biocarta***  ***2016*** | Reversal of Insulin Resistance by Leptin Homo sapiens h leptinPathway | 0.02313908479965613 | PRKAG1 |
|  | Rho-Selective Guanine Exchange Factor AKAP13 Mediates Stress Fiber Formation Homo sapiens h akap13Pathway | 0.02313908479965613 | PRKAG1 |
|  | Protein Kinase A at the Centrosome Homo sapiens h akapCentrosomePathway | 0.02313908479965613 | PRKAG1 |
|  | AKAP95 role in mitosis and chromosome dynamics Homo sapiens h akap95Pathway | 0.02313908479965613 | PRKAG1 |
|  | Control of Gene Expression by Vitamin D Receptor Homo sapiens h vdrPathway | 0.03549255368861455 | NCOR1 |
|  | ChREBP regulation by carbohydrates and cAMP Homo sapiens h chrebpPathway | 0.04356331810553544 | PRKAG1 |
|  | Mechanism of Gene Regulation by Peroxisome Proliferators via PPARa Homo sapiens h pparaPathway | 0.048281885719464374 | NCOR1 |
| **Elsevier** | Single-Strand Mismatch | 0.0471327929994943 | PMS1 |
|  | Single Strand Mismatch DNA Repair Suppression in Cancer | 0.0471327929994943 | PMS1 |
|  | Histone Acetylation | 0.0471327929994943 | NCOR1 |
|  | CD157 -> cADPR/Calcium Signaling | 0.0471327929994943 | RYR1 |
|  | beta-Cell Granules Exocytois | 0.0471327929994943 | RIMS2 |
|  | Myeloid Cell Differentiation Block in Leukemia | 0.0471327929994943 | NCOR1 |
|  | CD38 -> cADPR/Calcium Signaling | 0.0471327929994943 | RYR1 |
|  | Histone Methylation | 0.0471327929994943 | NCOR1 |
|  | RARA Signaling in Acute Myeloid Leukemia (M3) | 0.0471327929994943 | NCOR1 |
|  | Thyroid Hormones Common Genomic Effects in Hyperthyroidism | 0.0471327929994943 | NCOR1 |
|  | High Fat Diet Activates cAMP Related Exocytosis in Synapse and Endocrine Cell | 0.0471327929994943 | RIMS2 |
|  | Proteins with Altered Expression in Cancer-Associated Dysregulated DNA Repair | 0.0471327929994943 | PMS1 |
|  | Genes with Mutation in Cancer-Associated Dysregulated DNA Repair | 0.0471327929994943 | PMS1 |
|  | Huntingtin Gene Mutation in Striatal Neuron | 0.0471327929994943 | NCOR1 |
|  | Cochlear Hair Cell Stereocilia Proteins Mutations (Congenital Deafness) | 0.0471327929994943 | OTOA |
|  |  |  |  |
|  |  |  |  |
|  |  |  |  |
|  |  |  |  |
|  |  |  |  |
|  |  |  |  |
|  |  |  |  |
|  |  |  |  |
|  |  |  |  |
|  |  |  |  |
|  |  |  |  |
|  |  |  |  |
|  |  |  |  |
|  |  |  |  |

**Table 6.** Significant signaling pathway in male upregulated genes in All studies
