# Supplementary material for: Sex-biased transcriptomic landscapes in bipolar disorder: integrating neurobiology and clinical heterogeneity through cross-study meta-analysis
Source: Biol Sex Differ. 2026 May 8;17:125. doi: 10.1186/s13293-026-00870-4 (PMC13321544; doi:10.1186/s13293-026-00870-4)
Supplement: Supplementary file 12 — Supplementary Material 12 [file 13293_2026_870_MOESM12_ESM.docx]

**Table11**. Significant signaling pathway in male upregulated genes in striatal system

| Category | Pathway | Adjusted p-  value | Genes in the  pathway |
| --- | --- | --- | --- |
| [**BioPlanet 2019**](https://maayanlab.cloud/Enrichr/enrich) | Cytochrome P450 pathway | 0.04 | CYP27B1;CYP4V2;CYP1A1;CYP4B1;CYP7B1 |
| [**WikiPathways 2024 Human**](https://maayanlab.cloud/Enrichr/enrich) | Oxidation By Cytochrome P450 WP43 | 0.0001 | CYP27B1;CYP4V2;CYP1A1;CYP4B1;CYP7B1 |
| **Reactome**  **2024** | Cytochrome P450 - Arranged by Substrate Type | 0.04 | CYP27B1;CYP4V2;CYP1A1;CYP4B1;CYP7B1 |
|  | Phase I - Functionalization of Compounds | 0.04 | CYP27B1;CYP4V2;CYP1A1;CYP4B1;CYP7B1;CES2 |

| Category | Pathway | Adjusted p-  value | Genes in the  pathway |
| --- | --- | --- | --- |
| [**Reactome Pathways 2024**](https://maayanlab.cloud/Enrichr/enrich) | CDC42 GTPase Cycle | 1.7137604276440875E-5 | CPNE8;FMNL1;ARHGEF4;KCTD3;SRGAP3;ARHGEF5;FGD1;ARFGAP2;ARHGEF6;FGD2 |
|  | RHO GTPase Cycle | 0.003 | CPNE8;ANKLE2;FMNL1;COPS2;ARHGEF4;KCTD3;SRGAP3;ARHGEF5;FGD1;ARFGAP2;ARHGEF6;FGD2 |
|  | NRAGE Signals Death Through JNK | 0.003 | ARHGEF4;ARHGEF5;FGD1;ARHGEF6;FGD2 |
|  | Cell Death Signalling via NRAGE, NRIF and NADE | 0.009 | ARHGEF4;ARHGEF5;FGD1;ARHGEF6;FGD2 |
|  | G Alpha (12 13) Signalling Events | 0.009 | ARHGEF4;ARHGEF5;FGD1;ARHGEF6;FGD2 |
|  | P75 NTR Receptor-Mediated Signalling | 0.02 | ARHGEF4;ARHGEF5;FGD1;ARHGEF6;FGD2 |
| [**BioPlanet 2019**](https://maayanlab.cloud/Enrichr/enrich) | BRCA1, BRCA2 and ATR roles in cancer susceptibility | 0.03 | BRCA1;BRCA2;FANCE |
|  | Fanconi anemia pathway | 0.03 | BRCA1;BRCA2;FANCE |
| [**BioCarta 2016**](https://maayanlab.cloud/Enrichr/enrich) | Role of BRCA1, BRCA2 and ATR in Cancer Susceptibility Homo sapiens h atrbrcaPathway | 0.004 | BRCA1;BRCA2;FANCE |
|  | BRCA1-dependent Ub-ligase activity Homo sapiens h bard1Pathway | 0.007 | BRCA1;FANCE |

**Table12-significant signaling pathway in female upregulated genes in striatal system**
