# Supplementary material for: Sex-biased transcriptomic landscapes in bipolar disorder: integrating neurobiology and clinical heterogeneity through cross-study meta-analysis
Source: Biol Sex Differ. 2026 May 8;17:125. doi: 10.1186/s13293-026-00870-4 (PMC13321544; doi:10.1186/s13293-026-00870-4)
Supplement: Supplementary file 14 — Supplementary Material 14 [file 13293_2026_870_MOESM14_ESM.docx]

| 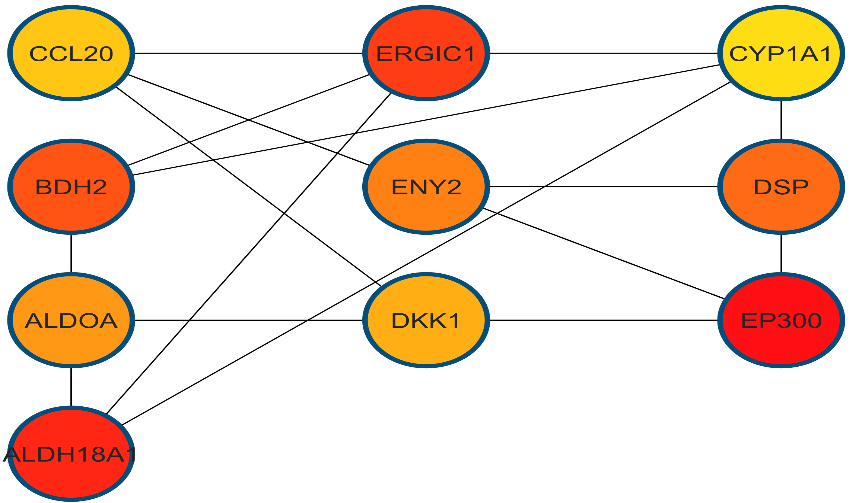B) Betweenness Centrality in the male striatal system | 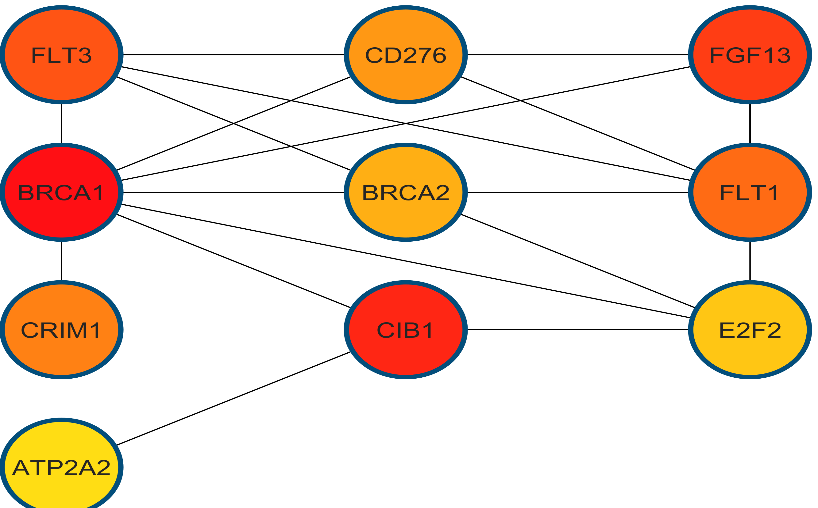A) Closeness Centrality in the male striatal system |
| --- | --- |
| 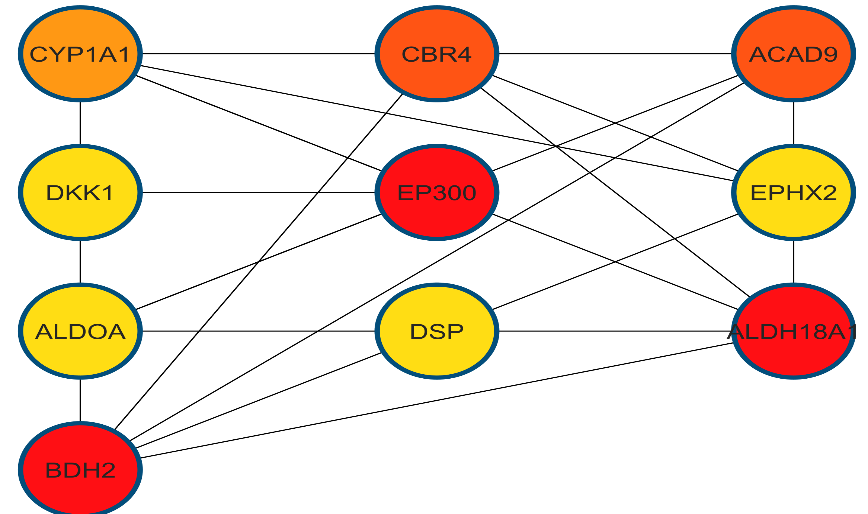D)Degree Centrality in the male striatal system | 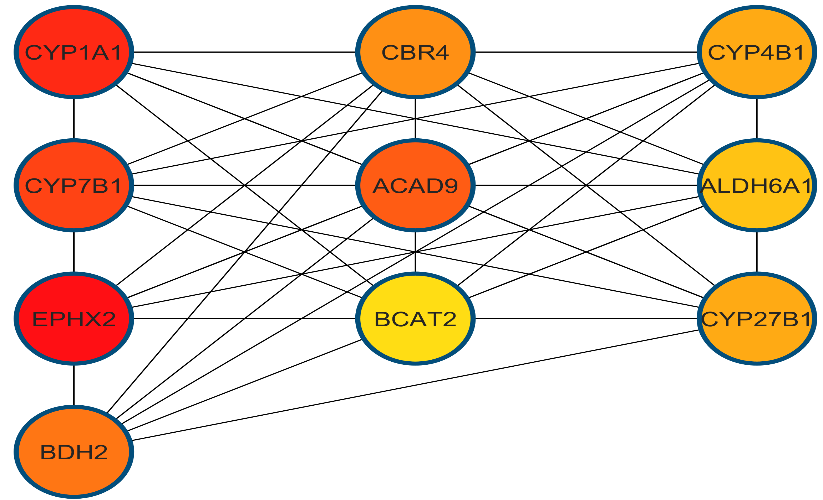C)Maximal Clique Centrality in the male striatal system |


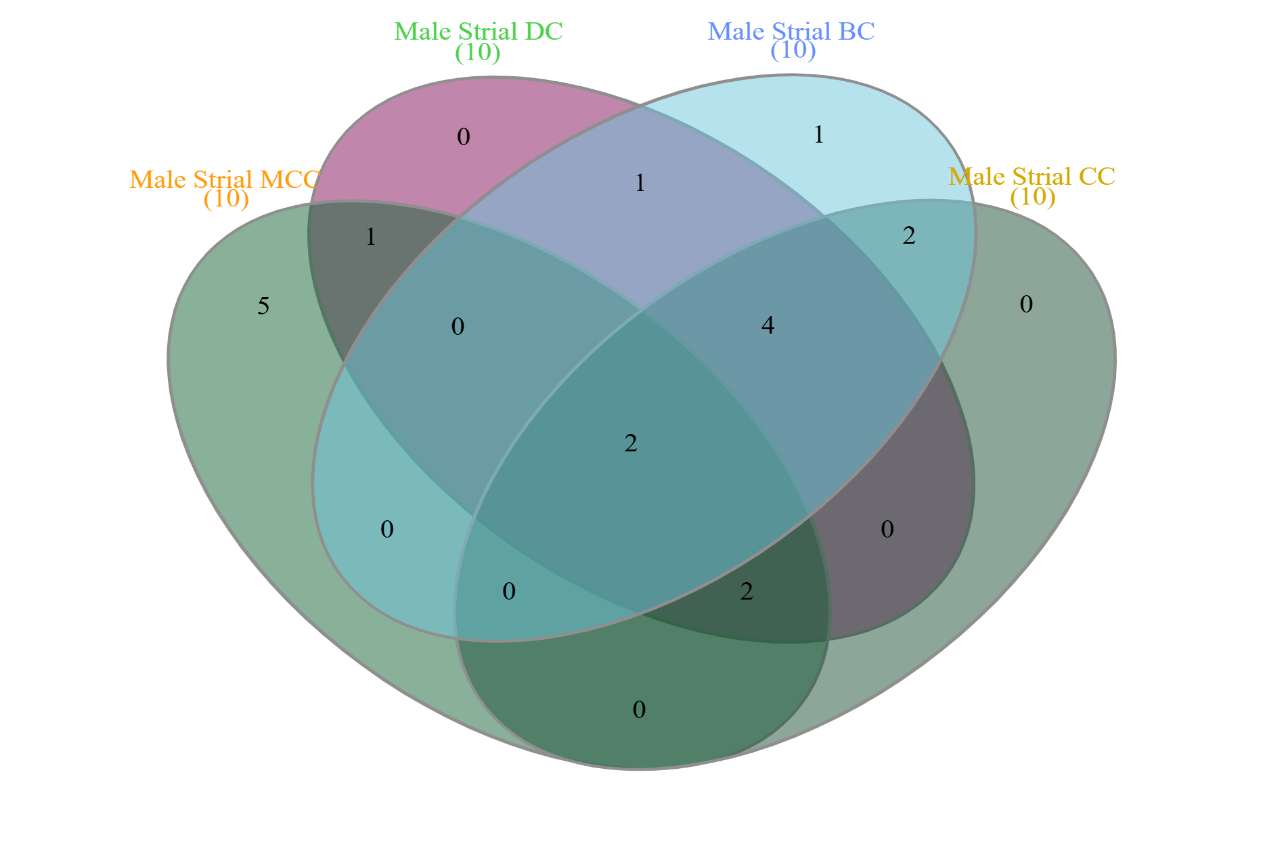


E) Overlap of top candidate hub genes identified using four distinct centrality measures in the male striatal system
