# Supplementary figures and images for: Sex-biased transcriptomic landscapes in bipolar disorder: integrating neurobiology and clinical heterogeneity through cross-study meta-analysis
Source: Biol Sex Differ. 2026 May 8;17:125. doi: 10.1186/s13293-026-00870-4 (PMC13321544; doi:10.1186/s13293-026-00870-4)

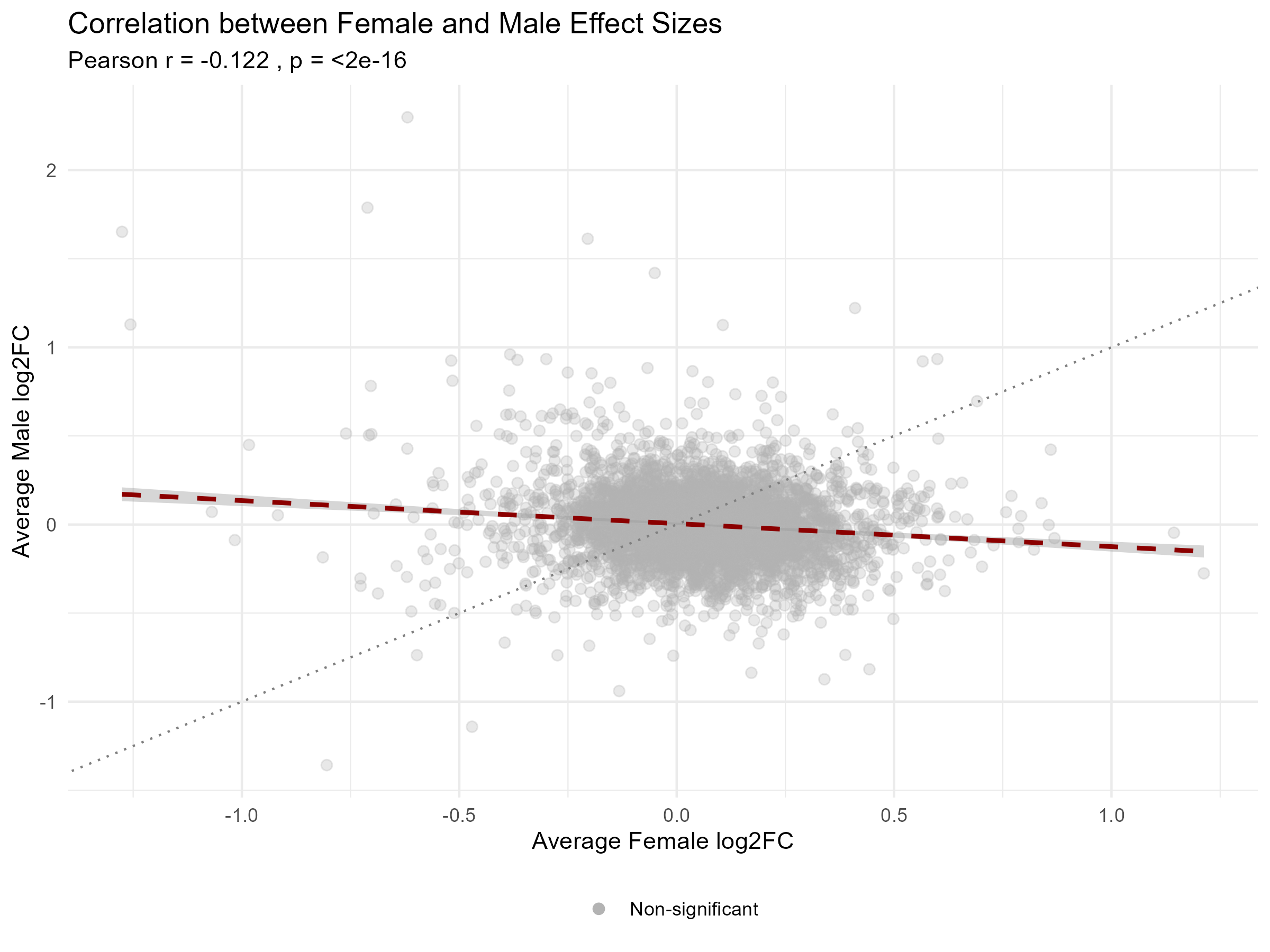

Supplement: Supplementary file 28 — Supplementary Material 28 [file 13293_2026_870_MOESM28_ESM.png]

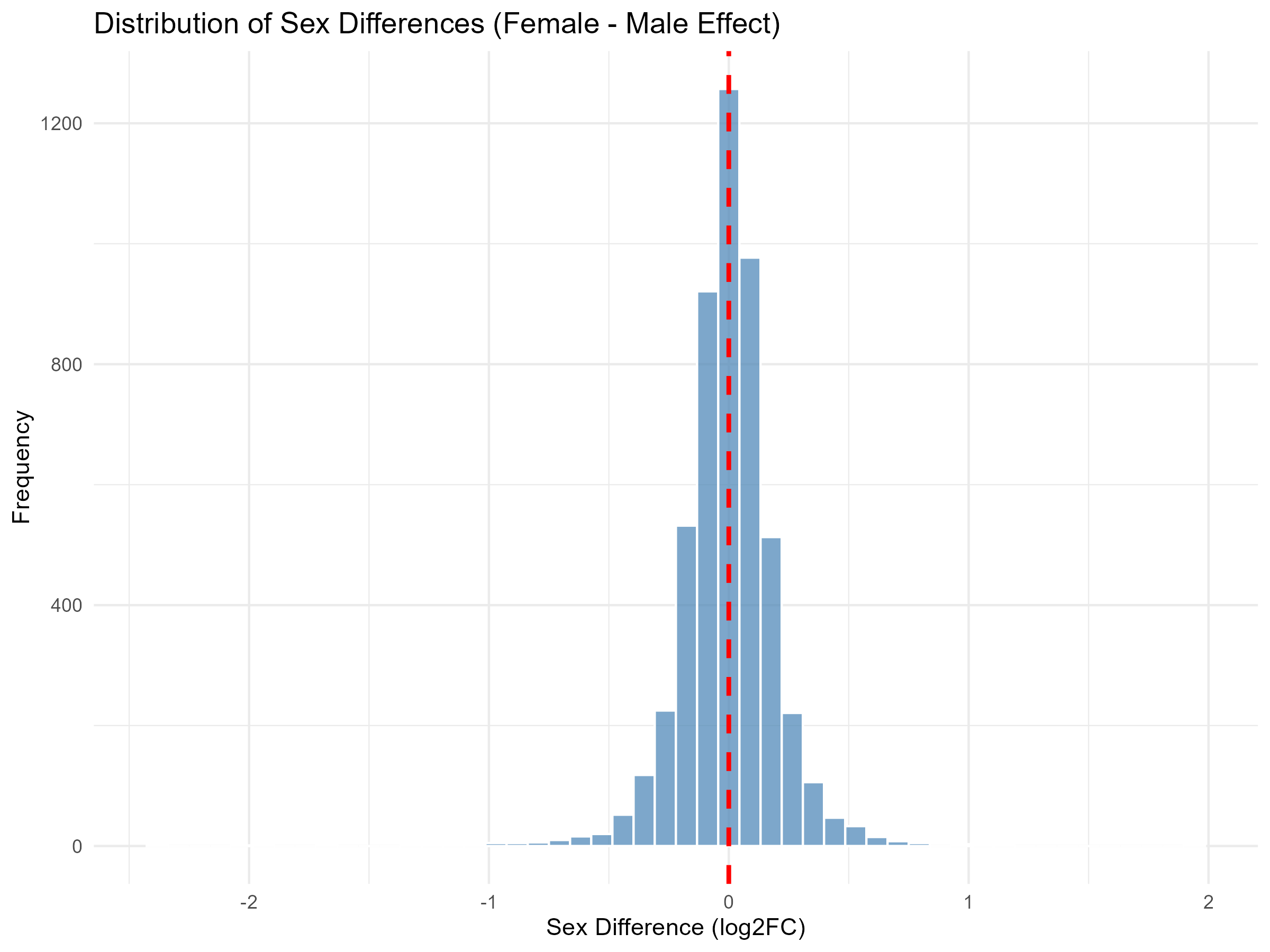

Supplement: Supplementary file 29 — Supplementary Material 29 [file 13293_2026_870_MOESM29_ESM.png]

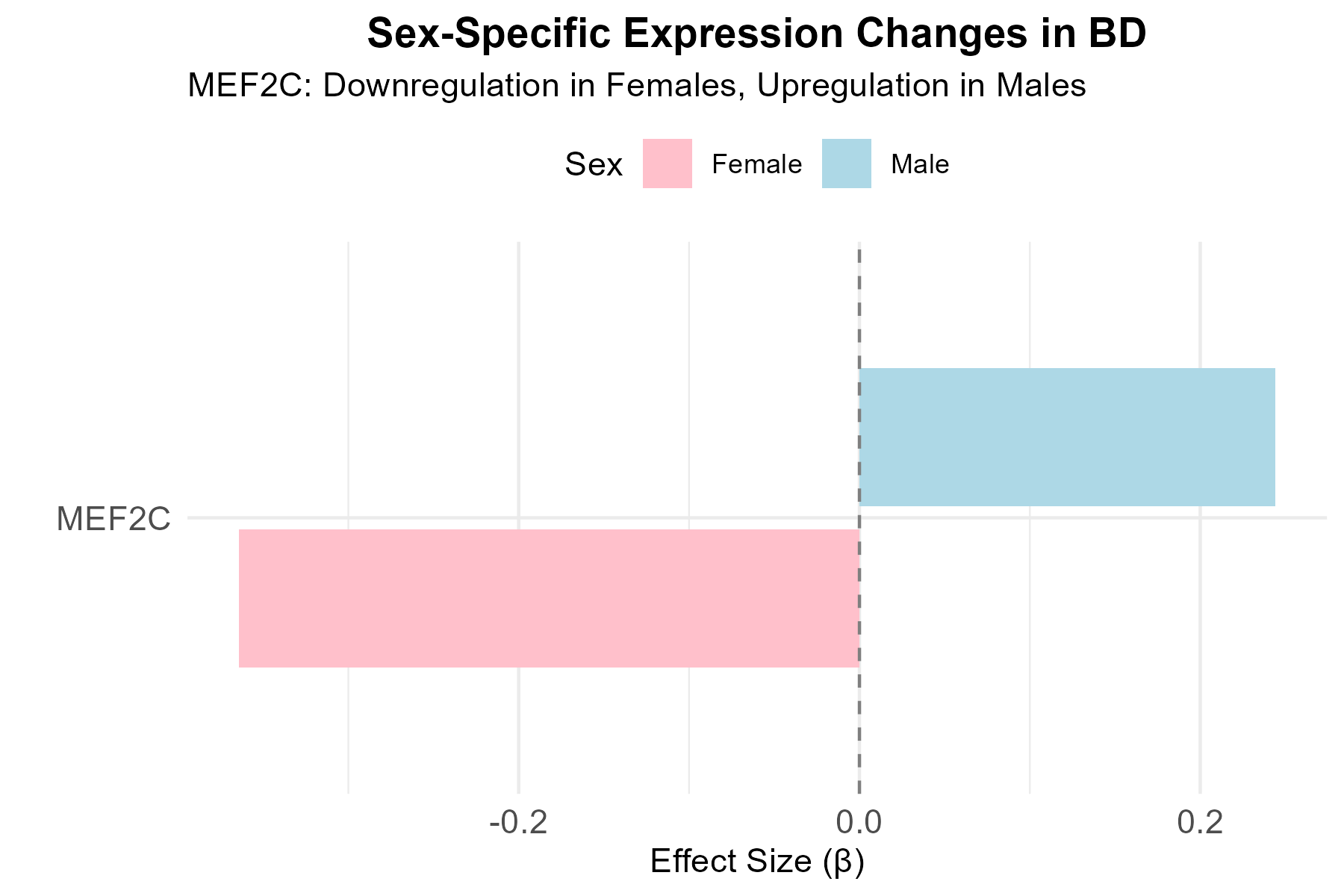

Supplement: Supplementary file 30 — Supplementary Material 30 [file 13293_2026_870_MOESM30_ESM.png]
